# Supplementary material for: Did the COVID-19 pandemic affect levels of burnout, anxiety and depression among doctors and nurses in Bangladesh? A cross-sectional survey study
Source: BMJ Open. 2024 Mar 7;14(3):e079350. doi: 10.1136/bmjopen-2023-079350 (PMC10921535; doi:10.1136/bmjopen-2023-079350)
Supplement: Author's manuscript [file bmjopen-2023-079350.draft_revisions.pdf]

BMJ Open

BMJ Open is committed to open peer review. As part of this commitment we make the peer review history of every article we publish publicly available.

When an article is published we post the peer reviewers' comments and the authors' responses online. We also post the versions of the paper that were used during peer review. These are the versions that the peer review comments apply to.

The versions of the paper that follow are the versions that were submitted during the peer review process. They are not the versions of record or the final published versions. They should not be cited or distributed as the published version of this manuscript.

BMJ Open is an open access journal and the full, final, typeset and author-corrected version of record of the manuscript is available on our site with no access controls, subscription charges or pay-per-view fees (http://bmjopen.bmj.com).

If you have any questions on BMJ Open's open peer review process please email info.bmjopen@bmj.com

BMJ Open

Burnout, anxiety and depression amongst healthcare workers in Bangladesh during the COVID-19 pandemic

Journal:	*BMJ Open*
Manuscript ID	bmjopen-2023-079350
Article Type:	Original research
Date Submitted by the Author:	04-Sep-2023

Complete List of Authors:	Hutchings, Hayley; Swansea University<br>Rahman, Mesbah; Swansea Bay University Health Board<br>Carter, Kymberley; Swansea University<br>Islam, Saiful; Swansea University<br>O'Neill, Claire; Swansea University<br>Roberts, Stephen; Swansea University<br>John, Ann; Swansea University<br>Fegan, Greg; Swansea University<br>Dave, Umakant; Swansea Bay University Health Board<br>Hawkes, Neil; Cwm Taf Morgannwg University Health Board<br>Ahmed, Faruque; Sheikh Russel National Gastroliver Institute and Hospital, Department of Gastroenterology<br>Hasan, Mahmud; Gastroliver Foundation<br>Azad, Abul Kalam; Dhaka Medical College and Hospital<br>Rahman, Md.; Dhaka Medical College and Hospital<br>Golam Kibria, Md; Sheikh Russel National Gastroliver Institute<br>Rahman, M. Masudur ; Sheik Russel National Gastroliver Institute<br>Mia, Titu; Mugda Medical College and Hospital<br>Akhter, Mahfuza; Mugda Medical College and Hospital<br>Williams, John; Swansea University, Faculty of Medicine, Health and Life Science

Keywords:	COVID-19, Anxiety disorders < PSYCHIATRY, Depression & mood disorders < PSYCHIATRY, Burnout, Professional

SCHOLARONE™
Manuscripts

I, the Submitting Author has the right to grant and does grant on behalf of all authors of the Work (as defined in the below author licence), an exclusive licence and/or a non-exclusive licence for contributions from authors who are: i) UK Crown employees; ii) where BMJ has agreed a CC-BY licence shall apply, and/or iii) in accordance with the terms applicable for US Federal Government officers or employees acting as part of their official duties; on a worldwide, perpetual, irrevocable, royalty-free basis to BMJ Publishing Group Ltd ("BMJ") its licensees and where the relevant Journal is co-owned by BMJ to the co-owners of the Journal, to publish the Work in this journal and any other BMJ products and to exploit all rights, as set out in our licence.

The Submitting Author accepts and understands that any supply made under these terms is made by BMJ to the Submitting Author unless you are acting as an employee on behalf of your employer or a postgraduate student of an affiliated institution which is paying any applicable article publishing charge ("APC") for Open Access articles. Where the Submitting Author wishes to make the Work available on an Open Access basis (and intends to pay the relevant APC), the terms of reuse of such Open Access shall be governed by a Creative Commons licence – details of these licences and which Creative Commons licence will apply to this Work are set out in our licence referred to above.

Other than as permitted in any relevant BMJ Author's Self Archiving Policies, I confirm this Work has not been accepted for publication elsewhere, is not being considered for publication elsewhere and does not duplicate material already published. I confirm all authors consent to publication of this Work and authorise the granting of this licence.

For peer review only

Burnout, anxiety and depression amongst healthcare workers in Bangladesh during the COVID-19 pandemic

Hayley A Hutchings[1]*, Mesbah Rahman[2], Kymberley Carter[1], Saiful Islam[1], Claire O'Neill[1], Stephen Roberts[1], Ann John[1], Greg Fegan[1], Umakant Dave[2], Neil Hawkes[3], Faruque Ahmed[4], Mahmud Hasan[5], Abul Kalam Azad[6], Mujibur Rahman[6], Md Golam Kibria[7], Masudur Rahman[7], Titu Mia[8], Mahfuza Akhter[8], John G Williams

Author Affiliations

[1]Swansea University Medical School, Faculty of Medicine, Health and Life Science, Swansea University, Swansea, United Kingdom.

[2]Swansea Bay University Health Board, Swansea, United Kingdom.

[3]Cwm Taf Morgannwg University Health Board, Abercynon, United Kingdom.

[4] Department of Gastroenterology, Sheikh Russel National Gastroliver Institute and Hospital, Dhaka, Bangladesh.

[5] Gastroliver Foundation, Dhaka, Bangladesh.

[6]Dhaka Medical College and Hospital, Dhaka, Bangladesh.

[7]Sheikh Russel National Gastroliver Institute, Dhaka, Bangladesh.

[8]Mugda Medical College and Hospital, Dhaka, Bangladesh.

*Corresponding author

Email: h.a.hutchings@swansea.ac.uk

For peer review only

For peer review only - http://bmjopen.bmj.com/site/about/guidelines.xhtml

Abstract

Introduction

Anecdotally, COVID-19 has caused severe disruption to clinical services in Bangladesh but the extent of this, and the impact on the diagnosis and management of other disorders, and on healthcare professionals is unclear. We aimed to assess perceived levels of anxiety, depression and burnout among healthcare workers during the COVID-19 pandemic in Bangladesh.

Methods

We undertook an online survey using RedCap, directed at doctors and nurses across four institutions in Bangladesh (The Sheikh Russel Gastro Liver Institute & Hospital (SRNGIH), Dhaka Medical College Hospital (DMCH), Mugda Medical College Hospital (MMCH), and M Abdur Rahim Medical College (MARMC) hospital. We collected information on demographics, access to wellbeing services, covid-related workload, as well anxiety, depression and burnout using two validated questionnaires (Hospital Anxiety and Depression Scale (HADS); Maslach Burnout Inventory (MBI).

Results

Of the 3000 participants approached, we received responses from 2705 (90.2%). There was a statistically significant difference in anxiety, depression and burnout scores across institutions (P< 0.01). Anxiety, depression and burnout scores were statistically worse in covid active staff compared with those not working on covid activities (P< 0.01 for HADS anxiety and depression and MBI EE, DP and PA). Over half of the participants exhibited some level of anxiety (SRNGIH- 52.2%; DMCH- 53.9%; MMCH- 61.3%; MARMC- 68%) with a high proportion experiencing depression (SRNGIH- 39.5%; DMCH- 38.7%; MMCH- 53.7%; MARMC- 41.1%).

For peer review only - http://bmjopen.bmj.com/site/about/guidelines.xhtml

For peer review only

Although mean burnout scores were within the normal range for each institution, a high proportion of staff (almost 20% in some instances) were shown to be classified as experiencing burnout by their EE, DP and PA scores.

Conclusion

We identified a high prevalence of perceived anxiety, depression and burnout among healthcare workers during the COVID-19 pandemic. This was worse in staff engaged in covid related activities.

288 words

For peer review only

For peer review only - http://bmjopen.bmj.com/site/about/guidelines.xhtml

Word count- 2329

Key words

Covid-19; health personnel; anxiety; depression; burnout, professional

What is already known on this topic

Although research in other countries has documented the impact of COVID-19 on healthcare workers, we are not aware of any studies that have explored this in Low and Middle Income Countries (LMIC).

What this study adds

We identified that a high proportion of medical personnel in Bangladesh were experiencing some level of anxiety and/or depression and that in some cases almost 20% reached the cut-off level for burnout.

How this study might affect research, practice or policy

Healthcare organisations need to consider how to plan for large-scale events such as future pandemics to ensure that staff can be appropriately supported during such times whilst maintaining delivery of high-quality healthcare.

For peer review only

Introduction

Bangladesh is a Low and/or Middle Income Countries (LMIC) country of 171 million people and a population density of 1,115.62 people per square kilometre, which ranks 10th in the world[1]. The health system of Bangladesh is a pluralistic system with four key sectors: government, private sector, nongovernmental organizations (NGOs) and donor agencies[2]. Bangladesh's economy has been growing with a GDP growth rate of more than 7.5%, however, about 20% of the population is poor[3]. Bangladesh has only 5.3 doctors per 10,000 people, 0.3 nurses per 1,000 people, 0.87 hospital beds per 1,000 people, 0.72 ICU beds and 1.1 ventilators per 100,000 people[3].

On January 30th, 2020 the World Health Organisation (WHO) declared the outbreak of COVID-19 a Public Health Emergency of International Concern[4]. According to the WHO COVID-19 is likely to have the biggest impact in countries with vulnerable health care systems. Due to its high population density, poor health care systems, poverty and a weak economy Bangladesh had the potential to be a risk candidate for poor outcomes from COVID-19[3 5]. Bangladesh reported their first case of COVID-19 on March 8, 2020[3].

The concept of 'staff burnout' was identified in 1974 by Freudenberger[6]. It was defined as "burnout in the individual's energy due to failure, wear-out, overloading, and unfulfilled desires". In 2019, 'burnout' was recognised by the World Health Organisation (WHO) as an 'occupational phenomenon' (World Health Organization classifies work "burnout" as an occupational phenomenon - CBS News), with a dedicated entry in its International Classification of Diseases Handbook (ICD-11 for Mortality and Morbidity Statistics (who.int)).

For peer review only - http://bmjopen.bmj.com/site/about/guidelines.xhtml

Since the beginning of the COVID-19 pandemic there has been extensive research exploring the impact of the pandemic on stress, anxiety, depression and burnout within the healthcare community. Researchers in Turkey, Saudi Arabia, United States, Canada, Romania, UK and Portugal have all demonstrated that COVID has had an impact on levels of burnout, stress and anxiety[7-14]. Factors such as worries about legal issues, age, experience, longer working hours, risk of exposing family members to COVID-19, direct COVID-facing roles, being redeployed, gender and coping strategies were related to levels of burnout and stress.

Anecdotally, COVID-19 has caused severe disruption to clinical services in Bangladesh but the extent of this, and the impact on the diagnosis and management of other disorders, and on healthcare professionals is unclear. Although research in other countries has documented the impact of COVID-19 on healthcare workers, we are not aware of any studies that have explored this in Low and Middle Income Countries (LMIC). Burnout has previously been explored in Bangladesh prior to the pandemic[15]. Qualitatively the pandemic has caused chaos, but there the impact on people and services has not yet been rigorously measured. The aim of this study was therefore to explore levels of burnout, anxiety and depression across healthcare workers within Bangladesh.

Materials and Methods

Study sites

A survey study was initiated in four large institutions within Bangladesh, three in Dhaka and one in Dinajpur (northern Bangladesh): The Sheikh Russel Gastro Liver Institute & Hospital

For peer review only - http://bmjopen.bmj.com/site/about/guidelines.xhtml

(SRNGIH), Dhaka Medical College Hospital (DMCH), Mugda Medical College Hospital (MMCH), and M Abdur Rahim Medical College (MARMC) hospital.

Study survey

The survey sought information on personal wellbeing and current access to local wellbeing services from healthcare staff working across all specialties at the four institutions. The total number of healthcare staff across the four institutions is 3000.

We used validated questionnaires to measure stress and anxiety (Hospital Anxiety and Depression Scale-HADS[16]) and burnout (Maslach Burnout Inventory- MBI[17] for Healthcare staff[18]). In addition to the two validated questionnaires, we asked participants at which institution they were based, their job/profession, their gender, their age, whether they were working on a COVID rota, whether they were a government employee, whether they undertook any private work and whether they were aware of any well-being services available to them. We also asked them if they had any difficulties answering any of the questions on the HADS or MBI. We provided participant information sheets and consent forms in Bengali and the HADS and MBI in English. Although there was a validated Bengali version of the HADS, there was no Bengali translation for the MBI. Therefore as most of the participants were English speaking, we opted to use English versions of both questionnaires. We provided additional guidance in Bengali to help participants complete these surveys (if required) and a study lead was available at each institution to provide further guidance, if necessary.

For peer review only - http://bmjopen.bmj.com/site/about/guidelines.xhtml

We invited staff working both in frontline COVID roles (i.e those directly managing COVID-19 patients) as well as those not to participate in the survey in order for comparisons to be made. Once participants had consented to take part, we provided the survey either on paper or as a link to an online survey via email. We collated the responses and they were entered onto an electronic data capture system REDCap by the study coordinator in Bangladesh. The REDCap database was hosted in Swansea and data analysis was performed by a Swansea based Bangladeshi statistician.

Public and Patient Involvement

As the study was designed as a staff survey exploring anxiety, depression and burnout in health care professional staff, it was not appropriate or possible to involve patients or the public in the design, or conduct, or reporting, or dissemination plans of our research.

For peer review only

For peer review only - http://bmjopen.bmj.com/site/about/guidelines.xhtml

Ethics approval

The study was approved by the Swansea University Medical School Research Ethics Committee as well as the Ethics Boards at each of the four institutions. We provided participants with an information sheet prior to them participating and all were required to sign a written consent form before proceeding with providing questionnaire responses for the survey. Information about participants was kept confidential and managed in accordance with the Data Protection Act, NHS Caldicott Guardian, The Research Governance Framework for Health and Social Care and Research Ethics Committee Approval.

Swansea University was the data controller and processor, and we provided training, guidance and practical support to the Bangladesh team, following General Data Protection Regulation (GDPR) guidelines. The project team in Swansea University who collated the data were all trained in GDPR. The only identifiable participant-level information was the signed consent forms for the survey which remained in Bangladesh. The paper questionnaires completed in Bangladesh were stored in a locked cabinet owned by one of the Dhaka site Principal Investigators. The consent forms were held separately to the survey data in different locked cabinet.

Analysis

For the validated surveys we calculated scores and used the standard processes to manage missing data in line with developers recommendations[16 19 20]. We explored the completeness of the data from all the surveys and sites and investigated any systematic errors and biases.

For peer review only - http://bmjopen.bmj.com/site/about/guidelines.xhtml

We looked for evidence of data being Missing Completely At Random (MCAR). In the analysis, the number and percentages of missing data are provided for all the outcomes.

The HADS anxiety and depression scores and the subscales of the MBI (emotional exhaustion (EE), depersonalization (DP), and personal accomplishment (PA)) are presented using summary statistics (mean and standard deviation (s.d.).

A higher score on the HADS indicates a worse anxiety/depression level. In terms of score interpretation, a HADS score of 0-7 is regarded as normal, 8-10 mild, 11-14 moderate and 15-21 severe[16]. Higher scores on the EE and DP subscales of the MBI indicate a higher burnout symptom burden; lower scores on the PA subscale indicate a higher burnout symptom burden[21]. We used previously published MBI raw score cut-points to assess burnout: ≥ 31, ≥ 14, and ≤ 29 respectively for the EE, DP, and PA subscales[21].

We used one-way ANOVA tests to compare anxiety, depressions and burnout scores across the different institutions. We used unpaired t- tests and chi-squared tests to compare scores from participants that actively worked on COVID frontline activities vs non-COVID frontline activities. A p value of less than 0.05 was regarded as significant. Analysis of the data was carried out using Stata™.

Results

Two thousand seven hundred and five (2705) participants out of 3000 (90.2%) responded to our survey. Of the 2705 responses, we received 400 (14.8%) from SRNGIH, 1283 (47.4%) from

For peer review only - http://bmjopen.bmj.com/site/about/guidelines.xhtml

DMCH, 628 (23.2%) from MMCH and 394 (14.6%) from MARMC. As a whole, 2108/2705 (77.9%) of the responses were from females and 592/2705 (21.9%) from males, with 5 (0.2%) responses where gender was not specified. Table 1 illustrates the demographic characteristics across the four institutions.

Table 2 illustrates the HADS scores across the four institutions. There was a statistically significant difference in HADS scores across the different institutions. The mean anxiety subscale score was in the moderate range for two institutions (DMCH and MMCH) and severe in the other two institutions. In terms of the mean depression subscale score three of the four institutions had depression scores within the normal range (SRNGIH, DMCH and MARMC), with the fourth institution (MMCH) having a mean score just above the normal range. Figure 1 illustrates the proportion of participants at each institution with normal (0-7), mild (8-10), or abnormal (11-14 moderate; 15-21 severe) HADS anxiety and depression scores. Over half of the participants exhibited some level of anxiety (SRNGIH- 52.2%; DMCH- 53.9%; MMCH- 61.3%; MARMC- 68%) with a high proportion experiencing depression (SRNGIH- 39.5%; DMCH- 38.7%; MMCH- 53.7%; MARMC- 41.1%).

Table 2 illustrates the Maslach scores across the four institutions. There was a statistically significant difference in scores across the four institutions. Mean EE, DP and PA scores did not indicate burnout. However, Figure 2 illustrates the proportion of staff at each institution who had scores that were classified as reaching the cut-off points that are classified as defining burnout for EE, DP and PA scores[21]. Therefore, although mean scores were within the normal range for each institution, a high proportion of staff (almost 20% in some instances) were shown to be classified as experiencing burnout EE, DP and PA by their scores.

For peer review only - http://bmjopen.bmj.com/site/about/guidelines.xhtml

For peer review only

Interestingly, the institution with the highest proportion of COVID-rota working (MMCH- 97%) had the highest HADS anxiety and depression scores. MMCH also had the highest MBI scores for the EE and DP subscales of all the institutions and the lowest MBI scores for PA, indicating higher levels of burnout as well as the most individuals achieving these burnout scores.

Table 3 illustrates the impact of a COVID frontline role on HADS and Maslach scores. Healthcare professionals who were engaged with frontline COVID duties had a statistically significantly higher anxiety and depression score when compared with healthcare professionals who were not engaged with COVID frontline duties. Similarly, Maslach scores for mean EE scores and DP scores were statistically significantly higher in staff with frontline COVID duties compared with staff not in frontline COVID duties indicating more burnout. Scores for Maslach personal accomplishment were lower in COVID duty staff than non-COVID duty staff, again indicating worse burnout.

For peer review only

For peer review only - http://bmjopen.bmj.com/site/about/guidelines.xhtml

Discussion

In this cross-sectional survey we found that a high proportion of staff working in the four hospitals had some level of anxiety (greater than 50% of participants) and depression (almost 40% of participants). Although the mean MBI scores for the EE, DP and PA scales were within the normal range, a high proportion of participants exceeded the thresholds defined as indicating burnout[21]. There were statistically significant differences across the four institutions for the HADS and MBI scores. We also found that those participants working on COVID activities had worse anxiety, depression and burnout scores that participants that were not working on COVID related activities. The differences in scores across institutions could be explained by the numbers of participants in each institution that were COVID active, as MMCH had the worse anxiety, depression and burnout scores and had the highest number of COVID active staff.

The prevalence of stress, anxiety and depression has been measured in previous studies undertaken throughout the pandemic period. Our findings concur with these studies where all have consistently identified a high prevalence of perceived stress, anxiety and depression among healthcare workers.[22 23]. A systematic umbrella review reported that there were differences in the prevalence of anxiety and depression amongst different healthcare workers, but that the prevalence was at least 17% and up to 40% in some groups[24]. As with our findings, frontline workers appear to be most affected by anxiety and depression[25].

A study in Bangladeshi nurses during the pandemic showed that depression was linked with burnout and that longer working hours contributed to higher depression[26]. The increased

For peer review only - http://bmjopen.bmj.com/site/about/guidelines.xhtml

workload associated with the pandemic has also been shown to result in higher levels of depression and burnout[27], findings that were also mirrored in our study.

Our study has a number of strengths. We had a large survey sample size of more than 2700 participants with an excellent response rate (90%) that surveyed multiple disciplines and different job roles in four major hospitals in Bangladesh. All information were collected anonymously which may have contributed to the willingness of participants to respond. There were some limitations. The survey tools themselves were administered in English as there was no validated Begali translation for one of the measures and because the majority of staff were English speaking. We did however provide some supplementary information in Begali to aid completion and added questions at the end of the survey asking for feedback about whether they had understood the survey questions. The responses returned indicated that there were no major problems with comprehension. The high response rate is also likely to indicate that most participants did not experience problems with completion. The survey itself was limited to doctors and nurses who are likely to have good levels of education. If the survey was rolled out to less qualified staff who may have poorer understanding of the English language, translation into their native language would be recommended.

Our findings suggest that working in the healthcare sector in busy and stressful situations has a negative impact on anxiety, depression and can increase levels of burnout. Healthcare organisations need to consider how to plan for large-scale events such as future pandemics to ensure that staff can be appropriately supported during such times whilst maintaining delivery of high-quality healthcare.

For peer review only - http://bmjopen.bmj.com/site/about/guidelines.xhtml

Acknowledgments

Jayne Price for her administrative support on the project, staff from Bangladesh involved in data collection/entry, all healthcare professionals who completed the survey.

For peer review only

For peer review only - http://bmjopen.bmj.com/site/about/guidelines.xhtml

References

1. Bangladesh Population [22 August 2022]. Available from: https://worldpopulationreview.com/countries/bangladesh-population.
2. Ahmed SM, Alam BB, Anwar I, et al. Bangladesh Health System Review, 2015.
3. Islam MT, Talukder AK, Siddiqui MN, et al. Tackling the COVID-19 pandemic: The Bangladesh perspective. *J Public Health Res* 2020;9(4):1794. doi: 10.4081/jphr.2020.1794 [published Online First: 20201014]
4. Sohrabi C, Alsafi Z, O'Neill N, et al. World Health Organization declares global emergency: A review of the 2019 novel coronavirus (COVID-19). *Int J Surg* 2020;76:71-76. doi: 10.1016/j.ijsu.2020.02.034 [published Online First: 20200226]
5. Md. Taimur Islam MT, Talukder AK, Badruzzaman M, et al. Health service facilities are positively linked with outcome of COVID-19 patients in majority of the countries: The global situation. *J Adv Biotechnol Exp Ther* 2020;3(4):36-41.
6. H.J. Freudenberger HL. Staff burn-out. *30* 1974;1:159-65.
7. Buran F, Altin Z. Burnout among physicians working in a pandemic hospital during the COVID-19 pandemic. *Leg Med (Tokyo)* 2021;51:101881. doi: 10.1016/j.legalmed.2021.101881 [published Online First: 2021/04/16]
8. AlJhani S, AlHarbi H, AlJameli S, et al. Burnout and coping among healthcare providers working in Saudi Arabia during the COVID-19 pandemic. *Middle East Current Psychiatry* 2021;28(29) doi: https://doi.org/10.1186/s43045-021-00108-6
9. Sasangohar F, Jones SL, Masud FN, et al. Provider Burnout and Fatigue During the COVID-19 Pandemic: Lessons Learned From a High-Volume Intensive Care Unit. *Anesth Analg* 2020;131(1):106-11. doi: 10.1213/ANE.0000000000004866 [published Online First: 2020/04/14]
10. Dillon EC, Stults CD, Deng S, et al. Women, Younger Clinicians', and Caregivers' Experiences of Burnout and Well-being During COVID-19 in a US Healthcare System. *J Gen Intern Med* 2022;37(1):145-53. doi: 10.1007/s11606-021-07134-4 [published Online First: 2021/11/04]
11. Khan N, Palepu A, Dodek P, et al. Cross-sectional survey on physician burnout during the COVID-19 pandemic in Vancouver, Canada: the role of gender, ethnicity and sexual orientation. *BMJ Open* 2021;11(5):e050380. doi: 10.1136/bmjopen-2021-050380 [published Online First: 2021/05/12]
12. Cotel A, Golu F, Pantea Stoian A, et al. Predictors of Burnout in Healthcare Workers during the COVID-19 Pandemic. *Healthcare (Basel)* 2021;9(3) doi: 10.3390/healthcare9030304 [published Online First: 2021/04/04]
13. Duarte I, Teixeira A, Castro L, et al. Burnout among Portuguese healthcare workers during the COVID-19 pandemic. *BMC Public Health* 2020;20(1):1885. doi: 10.1186/s12889-020-09980-z [published Online First: 2020/12/09]
14. Greene T, Harju-Seppänen J, Adeniji M, et al. Predictors and rates of PTSD, depression and anxiety in UK frontline health and social care workers during COVID-19. *European Journal of Psychotraumatology* 2021;12(1):1882781. doi: 10.1080/20008198.2021.1882781
15. Yasir Arafat SM, Kabir R. Burnout in Physicians: Global and Bangladesh Perspective. *EC Psychology and Psychiatry* 2017;2.3:112-17.
16. Zigmond AS, Snaith RP. The hospital anxiety and depression scale. *Acta Psychiatr Scand* 1983;67(6):361-70.
17. Maslach C, Jackson SE. The measurement of experienced burnout. *Journal of Occupational Behaviour* 1981;2:99-113.
18. Kalliath TJ, O'Driscoll MP, Gillespie DF, et al. A test of the Maslach burnout inventory in three samples of healthcare professionals *Work Stress*;14(1):35-50.

For peer review only - http://bmjopen.bmj.com/site/about/guidelines.xhtml

19. Maslach C, Jackson SE, Leiter MP. Maslach burnout inventory manual. 4th ed. Menlo Park, CA: Mind Garden Inc.
20. Stern AF. The hospital anxiety and depression scale. *Occup Med (Lond)* 2014;64(5):393-4. doi: 10.1093/occmed/kqu024
21. Brady KJS, Ni P, Sheldrick RC, et al. Describing the emotional exhaustion, depersonalization, and low personal accomplishment symptoms associated with Maslach Burnout Inventory subscale scores in US physicians: an item response theory analysis. *J Patient Rep Outcomes* 2020;4(1):42. doi: 10.1186/s41687-020-00204-x [published Online First: 20200601]
22. Aly HM, Nemr NA, Kishk RM, et al. Stress, anxiety and depression among healthcare workers facing COVID-19 pandemic in Egypt: a cross-sectional online-based study. *BMJ Open* 2021;11(4):e045281. doi: 10.1136/bmjopen-2020-045281 [published Online First: 2021/05/02]
23. Ali Sahebi A, Nejati-Zarnaqi B, Moayedi S, et al. The prevalence of anxiety and depression among healthcare workers during the COVID-19 pandemic: An umbrella review of meta-analyses. *Progress in Neuropsychopharmacology and Biological Psychiatry* 2021;107(110247)
24. Fernandez R, Sikhosana N, Green H, et al. Anxiety and depression among healthcare workers during the COVID-19 pandemic: a systematic umbrella review of the global evidence. *BMJ Open* 2021;11(9):e054528. doi: 10.1136/bmjopen-2021-054528 [published Online First: 2021/09/23]
25. Ahmed I, Asghar MS, Iqbal S, et al. Levels of Anxiety and Depression amongst the Frontline Healthcare Workers of COVID-19: A Cross-Sectional Survey with Follow-Up. *J Psychiatry Psychiatric Disord* 2020;4(4):270-84.
26. Chowdhury SR, Kabir H, Mazumder S, et al. Workplace violence, bullying, burnout, job satisfaction and their correlation with depression among Bangladeshi nurses: A cross-sectional survey during the COVID-19 pandemic. *PLoS One* 2022;17(9):e0274965. doi: 10.1371/journal.pone.0274965 [published Online First: 20220922]
27. Yin C, Ji J, Cao X, et al. Impact of long working hours on depressive symptoms among COVID-19 frontline medical staff: The mediation of job burnout and the moderation of family and organizational support. *Front Psychol* 2023;14:1084329. doi: 10.3389/fpsyg.2023.1084329 [published Online First: 20230215]

For peer review only - http://bmjopen.bmj.com/site/about/guidelines.xhtml

Figures

For peer review only

For peer review only - http://bmjopen.bmj.com/site/about/guidelines.xhtml

Figure 1. Proportion of participants in each institution with normal (0-7), mild (8-10) moderate (11-14) or severe (15-21): a) HADs Anxiety and b) HADs Depression scores.

a) HADs Anxiety scores

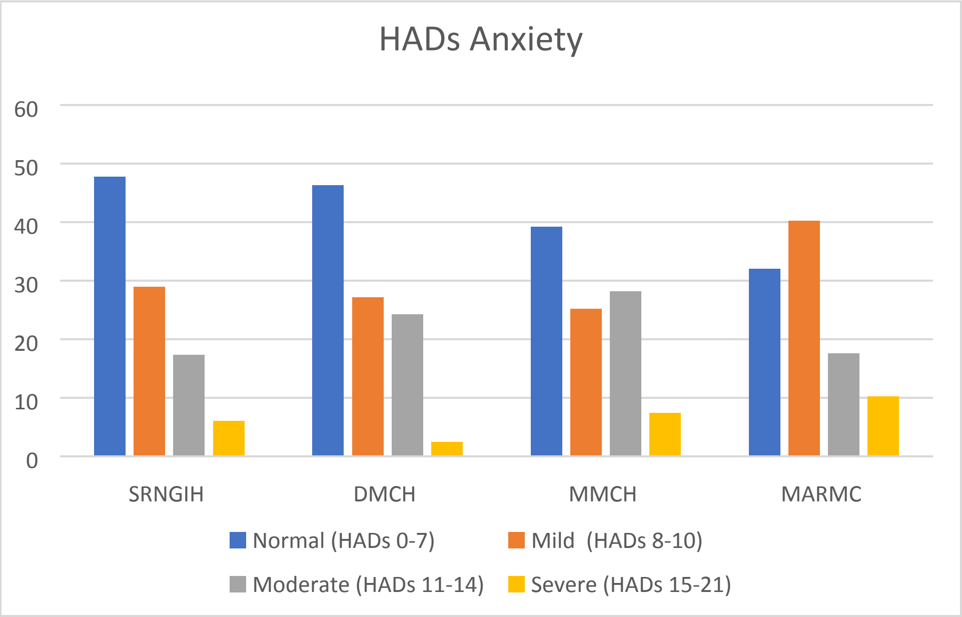

b) HADs Depression scores

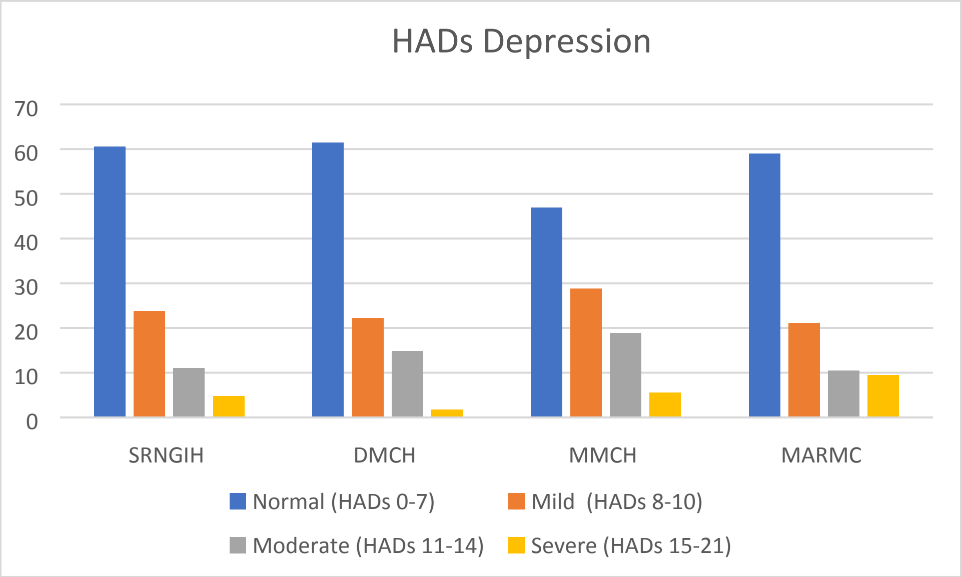

For peer review only - http://bmjopen.bmj.com/site/about/guidelines.xhtml

Figure 2. Proportion of participants in each institution achieving threshold levels for burnout (EE- greater than or equal to 31; DP- greater than or equal to 14; PA- less than or equal to 29)

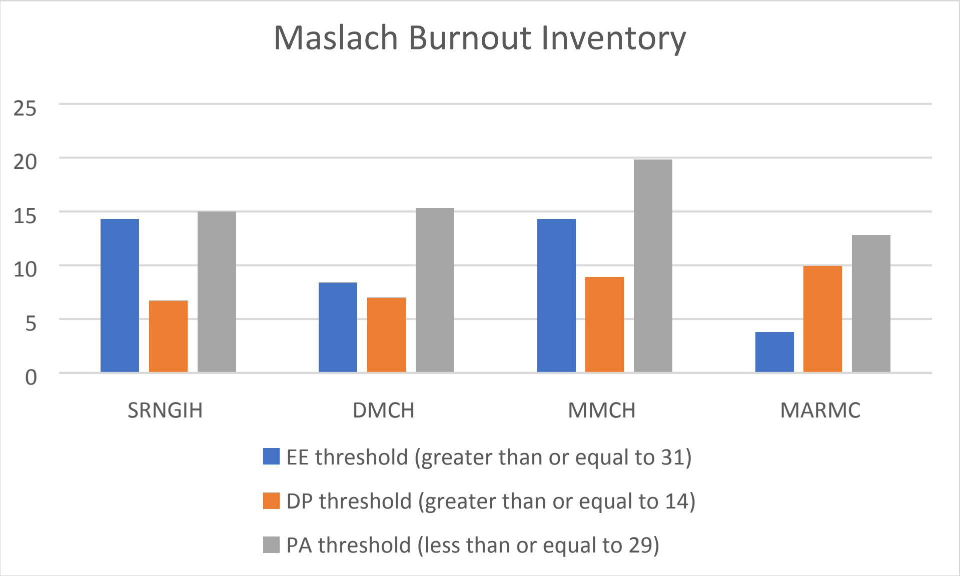

Maslach Burnout Inventory (MBI) %	SRNGIH	DMCH	MMCH	MARMC
EE threshold (greater than or equal to 31)	14.3	8.4	14.3	3.8
DP threshold (greater than or equal to 14)	6.7	7	8.9	9.9
PA threshold (less than or equal to 29)	15	15.3	19.8	12.8

For peer review only - http://bmjopen.bmj.com/site/about/guidelines.xhtml

Tables

For peer review only

For peer review only - http://bmjopen.bmj.com/site/about/guidelines.xhtml

Table 1: Demographic information by Institution

	Institution			
	**SRNGIH n=400**	**DMCH n=1283**	**MMCH n=628**	**MARMC n=394**
**Age (years)**				
Mean (s.d.)	32.2 (7.3)	33.6 (8.4)	37.1 (8.1)	32.3 (7.4)
**Gender (%)**				
Female: Male	72:28	82:18	71:29	83:17
**Departments (n,%)**				
Internal Medicine	12 (3)	227 (17.7)	99 (15.7)	57 (14.5)
Cardiology	1 (0.3)	1 (0.1)	12 (1.9)	26 (6.6)
Gastroenterology	207 (51.8)	6 (0.5)	9 (1.4)	0
Respiratory Medicine	4 (1.0)	7 (0.6)	8 (1.3)	0
Nephrology	1 (0.3)	55 (4.3)	20 (3.2)	16 (4.1)
Neurology	0	25 (1.9)	2 (0.3)	9 (2.3)
Rheumatology	3 (0.8)	3 (0.2)	5(0.8)	1 (0.3)
Surgery	21 (5.3)	280 (21.8)	69 (11.0)	83 (21.1)
Gynae/Obs	1 (0.3)	124 (9.7)	57 (9.1)	38 (9.6)
Psychiatry	1 (0.3)	8 (0.6)	4 (0.6)	0
Other	149 (37.3)	533 (41.5)	340 (54.1)	156 (39.6)
	0	14 (1.1)	3 (0.5)	8 (2.0)
**Professions (n,%)**				
Academic	31 (7.8)	2 (0.2)	119 (18.9)	5 (1.3)
Doctor	89 (22.3)	157 (12.2)	193 (30.7)	76 (19.3)
Nurse	277 (69.3)	1092 (85.1)	311 (49.5)	312 (79.2)
Other	3 (0.8)	30 (2.3)	4 (0.6)	1 (0.3)
Nmiss (%)	0	2 (0.2)	1 (0.2)	0
**COVID rota (n,%)**				
Yes	357 (89.3)	490 (38.2)	609 (97.0)	201 (51.0)
No	43 (10.8)	792 (61.7)	19 (3.0)	193 (49.0)
**Govt. Employee**				
Yes	395 (98.8)	1146 (89.3)	624 (99.4)	386 (98.0)
No	5 (1.3)	137 (10.7)	3 (0.5)	8 (2.0)
nmiss(%)	0	0	1 (0.2)	0
**Private Practice**				
Yes	76 (19.0)	29 (2.3)	117 (18.6)	8 (2.0)
No	324 (81.0)	1252 (97.6)	510 (81.2)	86 (98.0)
**Wellbeing Service**				
Yes	190 (47.5)	606 (47.2)	244 (38.9)	174 (44.2)
No	209 (52.3)	672 (52.4)	383 (60.9)	214 (54.3)
**Wellbeing Service (Getting help) [multiple response]**				
Friends/Family	20 (10.5)	63 (10.4)	48 (19.7)	4 (2.3)
Work Colleagues	93 (49.0)	356 (58.8)	110 (45.1)	120 (69.0)
Occupational Health	10 (5.3)	15 (2.5)	5 (2.1)	14 (8.1)
None	66 (34.7)	172 (28.4)	84 (34.4)	38 (21.8)
Other	2 (1.1)	9 (1.5)	0	0

For peer review only - http://bmjopen.bmj.com/site/about/guidelines.xhtml

Table 2: Hospital Anxiety and Depression Score (HADs) and Maslach Burnout Scores by Institution

	Institutions					
**HADs**	**SRNGIH**	**DMCH**	**MMCH**	**MARMC**	**F-test (*p-value*)**	**Tukey *t-test (significant)*[1]**
**Anxiety Subscale Score**<br>Mean (s.d.)	7.6 (4)	7.8 (4)	8.6 (4.1)	9 (4.3)	14.7 (<0.01)	G1 vs G2<br>G1 vs G3*<br>G1 vs G4*<br>G2 vs G3*<br>G2 vs G4*<br>G3 vs G4
**Depression Subscale Score**<br>Mean (s.d.)	6.3 (4.1)	6.6 (3.7)	7.9 (3.9)	6.9 (4.5)	17.3 (<0.01)	G1 vs G2<br>G1 vs G3*<br>G1 vs G4<br>G2 vs G3*<br>G2 vs G4<br>G3 vs G4*
**Maslach Subscale Scores**	**SRNGIH**	**DMCH**	**MMCH**	**MARMC**	**F-test (*p-value*)**	**Tukey *t-test (significant)*[1]**
**Emotional exhaustion (EE)**<br>Mean (s.d.)	17.5 (11.1)	15.4 (10.0)	19.1 (10.8)	15.7 (8.1)	20.6 (*p<0.01*)	G1 vs G2*<br>G1 vs G3<br>G1 vs G4<br>G2 vs G3*<br>G2 vs G4<br>G3 vs G4*
**Depersonalisation / loss of empathy (DP)**<br>Mean (s.d.)	4.3 (5.3)	4.3 (5.1)	5.2 (5.2)	4.7 (5.8)	4.11 (*p<0.01*)	G1 vs G2<br>G1 vs G3*<br>G1 vs G4<br>G2 vs G3*<br>G2 vs G4<br>G3 vs G4
**Personal accomplishment assessment (PA)**<br>Mean (s.d.)	39.0 (8.4)	38.5 (8.8)	36.5 (9.4)	37.8 (7.4)	8.9 (*p<0.01*)	G1 vs G2<br>G1 vs G3*<br>G1 vs G4<br>G2 vs G3*<br>G2 vs G4<br>G3 vs G4

For peer review only - http://bmjopen.bmj.com/site/about/guidelines.xhtml

Table 3: Hospital Anxiety and Depression Score (HADs) and Maslach Burnout Scores by COVID duty

	COVID Duty		*t*-test (*p*-value)
**HADs**	**Yes**	**No**	
**Anxiety Subscale Score**<br>Mean (s.d.)	8.3 (4.2)	7.8 (3.9)	3.4 (*p<0.01*)
**Depression Subscale Score**<br>Mean (s.d.)	7.1 (4.2)	6.5 (3.7)	4.2 (p<0.01)
**Maslach Subscale Scores**	**Yes**	**No**	**Chi-Square (*p-value*)**
**Emotional exhaustion (OE)**<br>Mean (s.d.)	17.5 (10.4)	15.3 (9.8)	5.4 (p<0.01)
**Depersonalisation / loss of empathy (DP)**<br>Mean (s.d.)	4.9 (5.4)	4.1 (5.0)	3.9 (p<0.01)
**Personal accomplishment assessment (PA)**<br>Mean (s.d.)	37.3 (9.1)	39.2 (8.1)	-5.4 (p<0.01)

For peer review only

BMJ Open

Did the COVID-19 pandemic affect levels of burnout, anxiety and depression among healthcare workers in Bangladesh?: A cross-sectional survey study

Journal:	*BMJ Open*
Manuscript ID	bmjopen-2023-079350.R1
Article Type:	Original research
Date Submitted by the Author:	05-Dec-2023
Complete List of Authors:	Hutchings, Hayley; Swansea University<br>Rahman, Mesbah; Swansea Bay University Health Board<br>Carter, Kymberley; Swansea University<br>Islam, Saiful; Swansea University<br>O'Neill, Claire; Swansea University<br>Roberts, Stephen; Swansea University<br>John, Ann; Swansea University<br>Fegan, Greg; Swansea University<br>Dave, Umakant; Swansea Bay University Health Board<br>Hawkes, Neil; Cwm Taf Morgannwg University Health Board<br>Ahmed, Faruque; Sheikh Russel National Gastroliver Institute and Hospital, Department of Gastroenterology<br>Hasan, Mahmud; Gastroliver Foundation<br>Azad, Abul Kalam; Dhaka Medical College and Hospital<br>Rahman, Md.; Dhaka Medical College and Hospital<br>Golam Kibria, Md; Sheikh Russel National Gastroliver Institute<br>Rahman, M. Masudur ; Sheik Russel National Gastroliver Institute<br>Mia, Titu; Mugda Medical College and Hospital<br>Akhter, Mahfuza; Mugda Medical College and Hospital<br>Williams, John; Swansea University, Faculty of Medicine, Health and Life Science
**Primary Subject Heading**:	Global health
Secondary Subject Heading:	Health services research, Mental health
Keywords:	COVID-19, Anxiety disorders < PSYCHIATRY, Depression & mood disorders < PSYCHIATRY, Burnout, Professional

SCHOLARONE™
Manuscripts

I, the Submitting Author has the right to grant and does grant on behalf of all authors of the Work (as defined in the below author licence), an exclusive licence and/or a non-exclusive licence for contributions from authors who are: i) UK Crown employees; ii) where BMJ has agreed a CC-BY licence shall apply, and/or iii) in accordance with the terms applicable for US Federal Government officers or employees acting as part of their official duties; on a worldwide, perpetual, irrevocable, royalty-free basis to BMJ Publishing Group Ltd ("BMJ") its licensees and where the relevant Journal is co-owned by BMJ to the co-owners of the Journal, to publish the Work in this journal and any other BMJ products and to exploit all rights, as set out in our licence.

The Submitting Author accepts and understands that any supply made under these terms is made by BMJ to the Submitting Author unless you are acting as an employee on behalf of your employer or a postgraduate student of an affiliated institution which is paying any applicable article publishing charge ("APC") for Open Access articles. Where the Submitting Author wishes to make the Work available on an Open Access basis (and intends to pay the relevant APC), the terms of reuse of such Open Access shall be governed by a Creative Commons licence – details of these licences and which Creative Commons licence will apply to this Work are set out in our licence referred to above.

Other than as permitted in any relevant BMJ Author's Self Archiving Policies, I confirm this Work has not been accepted for publication elsewhere, is not being considered for publication elsewhere and does not duplicate material already published. I confirm all authors consent to publication of this Work and authorise the granting of this licence.

For peer review only

Did the COVID-19 pandemic affect levels of burnout, anxiety and depression among healthcare workers in Bangladesh?: A cross-sectional survey study

Hayley A Hutchings[1]*, Mesbah Rahman[2], Kymberley Carter[1], Saiful Islam[1], Claire O'Neill[1], Stephen Roberts[1], Ann John[1], Greg Fegan[1], Umakant Dave[2], Neil Hawkes[3], Faruque Ahmed[4], Mahmud Hasan[5], Abul Kalam Azad[6], Mujibur Rahman[6], Md Golam Kibria[7], Masudur Rahman[7], Titu Mia[8], Mahfuza Akhter[8], John G Williams

Author Affiliations

[1]Swansea University Medical School, Faculty of Medicine, Health and Life Science, Swansea University, Swansea, United Kingdom.

[2]Swansea Bay University Health Board, Swansea, United Kingdom.

[3]Cwm Taf Morgannwg University Health Board, Abercynon, United Kingdom.

[4] Department of Gastroenterology, Sheikh Russel National Gastroliver Institute and Hospital, Dhaka, Bangladesh.

[5] Gastroliver Foundation, Dhaka, Bangladesh.

[6]Dhaka Medical College and Hospital, Dhaka, Bangladesh.

[7]Sheikh Russel National Gastroliver Institute, Dhaka, Bangladesh.

[8]Mugda Medical College and Hospital, Dhaka, Bangladesh.

*Corresponding author

Email: h.a.hutchings@swansea.ac.uk

For peer review only - http://bmjopen.bmj.com/site/about/guidelines.xhtml

Abstract

Introduction

COVID-19 has caused severe disruption to clinical services in Bangladesh but the extent of this, and the impact on healthcare professionals is unclear. We aimed to assess whether the COVID-19 pandemic had any impact on the perceived levels of anxiety, depression and burnout among healthcare workers.

Methods

We undertook an online survey using RedCap, directed at doctors and nurses across four institutions in Bangladesh (The Sheikh Russel Gastro Liver Institute & Hospital (SRNGIH), Dhaka Medical College Hospital (DMCH), Mugda Medical College Hospital (MMCH), and M Abdur Rahim Medical College (MARMC) hospital. We collected information on demographics, access to wellbeing services, covid-related workload, as well as anxiety, depression and burnout using two validated questionnaires (Hospital Anxiety and Depression Scale (HADS); Maslach Burnout Inventory (MBI)).

Results

Of the 3000 participants approached, we received responses from 2705 (90.2%). There was a statistically significant difference in anxiety, depression and burnout scores across institutions (P< 0.01). Anxiety, depression and burnout scores were statistically worse in covid active staff compared with those not working on covid activities (P< 0.01 for HADS anxiety and depression and MBI EE, DP and PA). Over half of the participants exhibited some level of anxiety (SRNGIH- 52.2%; DMCH- 53.9%; MMCH- 61.3%; MARMC- 68%) with a high proportion experiencing depression (SRNGIH- 39.5%; DMCH- 38.7%; MMCH- 53.7%; MARMC- 41.1%). Although mean burnout scores were within the normal range for each institution, a high

For peer review only - http://bmjopen.bmj.com/site/about/guidelines.xhtml

proportion of staff (almost 20% in some instances) were shown to be classified as experiencing burnout by their EE, DP and PA scores.

Conclusion

We identified a high prevalence of perceived anxiety, depression and burnout among healthcare workers during the COVID-19 pandemic. This was worse in staff engaged in covid related activities. These findings could help healthcare organisations to help plan for future similar events.

296 words

For peer review only

For peer review only - http://bmjopen.bmj.com/site/about/guidelines.xhtml

Word count- 2401

Key words

Covid-19; health personnel; anxiety; depression; burnout, professional

Strengths and Limitations of the study

- We conducted a large scale prospective cross-sectional survey using RedCap™, across four institutions in Bangladesh with nurses and doctors.
- We employed two validated measures to assess anxiety, depression and burnout.
- The study was operationalised by personnel based in Bangladesh, whilst the analysis was conducted remotely in the U.K.
- Further work is needed to determine whether anxiety, depression and burnout persist post-COVID.

For peer review only

For peer review only - http://bmjopen.bmj.com/site/about/guidelines.xhtml

Introduction

Bangladesh is a Low and/or Middle Income Countries (LMIC) country of 171 million people and a population density of 1,115.62 people per square kilometre, which ranks 10th in the world[1]. The health system of Bangladesh is a pluralistic system with four key sectors: government, private sector, nongovernmental organizations (NGOs) and donor agencies[2]. Bangladesh's economy has been growing with a GDP growth rate of more than 7.5%, however, a high proportion of the population (about 20%) are living below the poverty line[3, 4]. Bangladesh has only 5.3 doctors per 10,000 people, 0.3 nurses per 1,000 people, 0.87 hospital beds per 1,000 people, 0.72 ICU beds and 1.1 ventilators per 100,000 people[3].

On January 30th, 2020 the World Health Organisation (WHO) declared the outbreak of COVID-19 a Public Health Emergency of International Concern[5]. According to the WHO COVID-19 is likely to have the biggest impact in countries with vulnerable health care systems. Due to its high population density, poor health care systems, poverty and a weak economy Bangladesh had the potential to be a risk candidate for poor outcomes from COVID-19[3]. Bangladesh reported their first case of COVID-19 on March 8, 2020[3, 6, 7].

The concept of 'staff burnout' was identified in 1974 by Freudenberger[8]. It was defined as "burnout in the individual's energy due to failure, wear-out, overloading, and unfulfilled desires". In 2019, 'burnout' was recognised by the World Health Organisation (WHO) as an 'occupational phenomenon'[9], with a dedicated entry in its International Classification of Diseases Handbook (ICD-11 for Mortality and Morbidity Statistics (who.int)).

For peer review only - http://bmjopen.bmj.com/site/about/guidelines.xhtml

Since the beginning of the COVID-19 pandemic there has been extensive research exploring the impact of the pandemic on stress, anxiety, depression and burnout within the healthcare community[10]. Researchers in Turkey, Saudi Arabia, United States, Canada, Romania, UK and Portugal have all demonstrated that COVID has had an impact on levels of burnout, stress and anxiety[11-18]. Factors such as worries about legal issues, age, experience, longer working hours, risk of exposing family members to COVID-19, direct COVID-facing roles, being redeployed, gender and coping strategies were related to levels of burnout and stress.

COVID-19 has caused severe disruption in Bangladesh. This has included the country's finances, the delivery of clinical services and its impact on the people of Bangladesh[19-23]. Research in other countries has documented the impact of COVID-19 on healthcare workers, but most of this has been undertaken in higher income countries[11-18]. The potential impact that burnout could have from the Bangladesh perspective has previously been considered prior to the pandemic[24] and there has been some work exploring burnout and work stresses in nurses[25] and depression in trainee medical students[26] during the pandemic but there has been limited large-scale work exploring the impact of COVID- 19 on anxiety, depression and burnout in healthcare workers in Bangladesh. The aim of this study was therefore to explore levels of burnout, anxiety and depression across healthcare workers within Bangladesh. We hypothesised that those staff involved in COVID-19 frontline activities would have worse burnout, anxiety and depression than those staff not engaged in COVID-19 frontline activities.

Materials and Methods

Study sites

For peer review only - http://bmjopen.bmj.com/site/about/guidelines.xhtml

A cross-sectional prospective survey study was initiated between June and August 2021 in four large institutions within Bangladesh, three in Dhaka and one in Dinajpur (northern Bangladesh): The Sheikh Russel Gastro Liver Institute & Hospital (SRNGIH), Dhaka Medical College Hospital (DMCH), Mugda Medical College Hospital (MMCH), and M Abdur Rahim Medical College (MARMC) hospital.

Study survey

The survey sought information on personal wellbeing and current access to local wellbeing services from healthcare staff working across all specialties at the four institutions. The total number of healthcare staff across the four institutions is 3000. The study team included a local clinician at each of the four sites who acted as the site principal investigator (PI). Each PI sent an email on behalf of the study team to all clinical staff at their institution inviting them to participate.

We used validated questionnaires to measure stress and anxiety. We used the Hospital Anxiety and Depression Scale-HADS[27] to measure Anxiety and Depression. A higher score on the HADS indicates a worse anxiety/depression level. In terms of score interpretation, a HADS score of 0-7 is regarded as normal, 8-10 mild, 11-14 moderate and 15-21 severe[27].

We used the Maslach Burnout Inventory- MBI[28] for Healthcare staff[29] to measure burnout in the form of : Emotional Exhaustion (EE), Depersonalisation/loss of empathy (DP), and Personal Accomplishment (PA). Higher scores on the EE and DP subscales of the MBI indicate a higher burnout symptom burden; lower scores on the PA subscale indicate a higher

For peer review only - http://bmjopen.bmj.com/site/about/guidelines.xhtml

burnout symptom burden[30]. We used previously published MBI raw score cut-points to assess burnout: ≥ 31, ≥ 14, and ≤ 29 respectively for the EE, DP, and PA subscales[30].

In addition to the two validated questionnaires, we asked participants at which institution they were based, their job/profession, their gender, their age, whether they were working on a COVID rota, whether they were a government employee, whether they undertook any private work and whether they were aware of any well-being services available to them. We also asked them if they had any difficulties answering any of the questions on the HADS or MBI. We provided participant information sheets and consent forms in Bengali and the HADS and MBI in English. Although there was a validated Bengali version of the HADS, there was no Bengali translation for the MBI. Therefore as most of the participants were English speaking, we opted to use English versions of both questionnaires. We provided additional guidance in Bengali to help participants complete these surveys (if required) and the local PI was available at each institution to provide further guidance, if necessary.

We invited staff working both in frontline COVID roles (i.e those directly managing COVID-19 patients) as well as those not to participate in the survey in order for comparisons to be made. Once participants had consented to take part, we provided the survey either on paper or as a link to an online survey via email. We collated the responses and they were entered onto an electronic data capture system REDCap by the study coordinator in Bangladesh. The REDCap database was hosted in Swansea and data analysis was performed by a Swansea based Bangladeshi statistician.

Public and Patient Involvement

For peer review only - http://bmjopen.bmj.com/site/about/guidelines.xhtml

None

Analysis

For the validated surveys we calculated scores and used the standard processes to manage missing data in line with developers recommendations[27, 31, 32]. We explored the completeness of the data from all the surveys and sites and investigated any systematic errors and biases. We looked for evidence of data being Missing Completely At Random (MCAR). In the analysis, the number and percentages of missing data are provided for all the outcomes.

The HADS anxiety and depression scores and the subscales of the MBI (emotional exhaustion (EE), depersonalization (DP), and personal accomplishment (PA)) are presented using summary statistics (mean and standard deviation (s.d.).

We used one-way ANOVA F-tests to compare anxiety, depression and burnout scores across the different institutions. We used Tukey post hoc tests to identify where significant differences existed between different groups after the ANOVA test. We used unpaired t- tests and chi-squared tests to compare scores from participants that actively worked on COVID frontline activities vs non-COVID frontline activities. A p value of less than 0.05 was regarded as significant. Analysis of the data was carried out using Stata™.

Results

Two thousand seven hundred and five (2705) participants out of 3000 (90.2%) responded to our survey. Of the 2705 responses, we received 400 (14.8%) from SRNGIH, 1283 (47.4%) from

For peer review only - http://bmjopen.bmj.com/site/about/guidelines.xhtml

DMCH, 628 (23.2%) from MMCH and 394 (14.6%) from MARMC. As a whole, 2108/2705 (77.9%) of the responses were from females and 592/2705 (21.9%) from males, with 5 (0.2%) responses where gender was not specified. Table 1 illustrates the demographic characteristics across the four institutions.

Table 2 illustrates the HADS scores across the four institutions. There was a statistically significant difference in HADS scores across the different institutions ($p<0.05$). The mean anxiety subscale score was in the normal range (0-7) for two institutions (DMCH and MMCH) and in the mild range (8-10) in the other two institutions. In terms of the mean depression subscale score three of the four institutions had depression scores within the normal range (SRNGIH, DMCH and MARMC), with the fourth institution (MMCH) having a mean score just above the normal range. Figure 1 illustrates the proportion of participants at each institution with normal (0-7), mild (8-10), or abnormal (11-14 moderate; 15-21 severe) HADS anxiety and depression scores. Over half of the participants exhibited some level of anxiety (SRNGIH- 52.2%; DMCH- 53.9%; MMCH- 61.3%; MARMC- 68%) with a high proportion experiencing depression (SRNGIH- 39.5%; DMCH- 38.7%; MMCH- 53.7%; MARMC- 41.1%). There was a statistically significant difference in the proportion of participants experiencing anxiety or depression across the different institutions ($P<0.05$).

Table 2 illustrates the Maslach scores across the four institutions. There was a statistically significant difference in scores across the four institutions ($p<0.05$). Mean EE, DP and PA scores did not indicate burnout. However, Figure 2 illustrates the proportion of staff at each institution who had scores that were classified as reaching the cut-off points that are classified as defining burnout for EE, DP and PA scores[30]. Therefore, although mean scores were

For peer review only - http://bmjopen.bmj.com/site/about/guidelines.xhtml

within the normal range for each institution, a high proportion of staff (almost 20% in some instances) were shown to be classified as experiencing burnout EE, DP and PA by their scores. There was a statistically significant difference ($p<0.05$) in the proportion of participants by institution experiencing burnout as indicated by their EE and PA scores. There was however no significant difference in scores across institutions by DP scores ($p>0.05$).

As we expected the institution with the highest proportion of COVID-rota working (MMCH-97%) had the highest HADS anxiety and depression scores. MMCH also had the highest MBI scores for the EE and DP subscales of all the institutions and the lowest MBI scores for PA, indicating higher levels of burnout as well as the most individuals achieving these burnout scores.

Table 3 illustrates the impact of a COVID frontline role on HADS and Maslach scores. Healthcare professionals who were engaged with frontline COVID duties had a statistically significantly higher anxiety (8.3 vs 7.8; $p<0.01$) and depression (7.1 vs 6.5; $p<0.05$) scores when compared with healthcare professionals who were not engaged with COVID frontline duties. Similarly, Maslach scores for mean EE scores (17.5 vs 15.3; $p<0.05$) and DP scores (4.9 vs 4.1; $p<0.01$) were statistically significantly higher in staff with frontline COVID duties compared with staff not in frontline COVID duties indicating more burnout. Scores for Maslach personal accomplishment were lower (37.3 vs 39.2; $p<0.01$) in COVID duty staff than non-COVID duty staff, again indicating worse burnout.

For peer review only - http://bmjopen.bmj.com/site/about/guidelines.xhtml

Discussion

In this cross-sectional prospective survey we found that a high proportion of staff working in the four hospitals had some level of anxiety (greater than 50% of participants) and depression (almost 40% of participants). Although the mean MBI scores for the EE, DP and PA scales were within the normal range, a high proportion of participants exceeded the thresholds defined as indicating burnout[30]. There were statistically significant differences across the four institutions for the HADS and MBI scores. We also found that those participants working on COVID activities had worse anxiety, depression and burnout scores that participants that were not working on COVID related activities. The differences in scores across institutions could be explained by the numbers of participants in each institution that were COVID active, as MMCH had the worse anxiety, depression and burnout scores and had the highest number of COVID active staff.

The prevalence of stress, anxiety and depression has been measured in previous studies undertaken throughout the pandemic period. Our findings concur with these studies where all have consistently identified a high prevalence of perceived stress, anxiety and depression among healthcare workers.[33, 34]. A systematic umbrella review reported that there were differences in the prevalence of anxiety and depression amongst different healthcare workers, but that the prevalence was at least 17% and up to 40% in some groups[35]. As with our findings, frontline workers appear to be most affected by anxiety and depression[12].

A study in Bangladeshi nurses during the pandemic showed that depression was linked with burnout and that longer working hours contributed to higher depression[25]. The increased

For peer review only - http://bmjopen.bmj.com/site/about/guidelines.xhtml

workload associated with the pandemic has also been shown to result in higher levels of depression and burnout[36], findings that were also mirrored in our study. A recent pilot study in Bangladesh similarly identified a very high prevalence of depression symptoms among Bangladeshi medical students[26].

Our study has a number of strengths. We had a large survey sample size of more than 2700 participants with an excellent response rate (90%) that surveyed multiple disciplines and different job roles in four major hospitals in Bangladesh. All information were collected anonymously which may have contributed to the willingness of participants to respond. There were some limitations. The survey tools themselves were administered in English as there was no validated Bengali translation for one of the measures and because the majority of staff were English speaking. We did however provide some supplementary information in Bengali to aid completion and added questions at the end of the survey asking for feedback about whether they had understood the survey questions. The responses returned indicated that there were no major problems with comprehension. The high response rate is also likely to indicate that most participants did not experience problems with completion.

The survey itself was limited to doctors and nurses who are likely to have good levels of education. If the survey was rolled out to less qualified staff who may have poorer understanding of the English language, translation into their native language would be recommended. This survey was conducted during the second wave of COVID-19[37] when stresses on healthcare workers were likely to be high. Further work is needed to explore whether the levels of anxiety, depression and burnout still persist in Bangladesh post pandemic.

For peer review only - http://bmjopen.bmj.com/site/about/guidelines.xhtml

Our findings suggest that working in the healthcare sector in busy and stressful situations has a negative impact on anxiety, depression and can increase levels of burnout in healthcare workers in Bangladesh. This was worse if they had frontline COVID-19 duties.

Healthcare organisations need to consider how to plan for large-scale events such as future pandemics to ensure that staff can be appropriately supported during such times whilst maintaining delivery of high-quality healthcare.

Acknowledgments

Jayne Price for her administrative support on the project, staff from Bangladesh involved in data collection/entry, all healthcare professionals who completed the survey.

Contributorship Statement

HAH, JGW, CON, KC, GF and MR conceived and secured funding for the study. HAH was the study chief investigator. KC was the research manager and was responsible for overseeing and securing all governance approvals. SI led on data management and statistical analysis with oversight from GF. SR and AJ provided input on study design and interpretation. UD, NH and MR provided clinical input to the study. FA, MH, AK, MR, MGK, MasR, TM, MA were the Bangladesh principal investigators and were responsible for leading on institutional ethics approvals, site set-up and local recruitment and data collection. HAH led on the drafting of the manuscript. All reviewed and provided input on the final manuscript. HAH is

For peer review only - http://bmjopen.bmj.com/site/about/guidelines.xhtml

the guarantor for the manuscript and accepts full responsibility for the work and/or the conduct of the study, had access to the data and controlled the decision to publish.

Competing Interests

None

Funding

This study was funded by Swansea University through their Global Challenges Research Fund.

Grant number: N/A.

Data Sharing Statement

Data are available upon reasonable request.

For peer review only - http://bmjopen.bmj.com/site/about/guidelines.xhtml

Ethics approval

The study was approved by the Swansea University Medical School Research Ethics Sub-Committee (Ref: 2020-0070), as well as the Ethics Boards at each of the four institutions (Sheik Russel Gastroliver Institute and Hospital Institutional Ethical Review Committee (Ref: SRG&H/ERC/2020-2021/02); M Abdur Rahim Medical College Institutional Review Board (Ref: MARMCD/2021/370); Mugda Medical College & Hospital Institutional Review Board (no ref); Dhaka Medical College Institutional Review Board (Ref: ERC-DMC-ECC/2021/47)).

We provided participants with an information sheet prior to them participating and all were required to sign a written consent form before proceeding with providing questionnaire responses for the survey. Information about participants was kept confidential and managed in accordance with the Data Protection Act, NHS Caldicott Guardian, The Research Governance Framework for Health and Social Care and Research Ethics Committee Approval.

Swansea University was the data controller and processor, and we provided training, guidance and practical support to the Bangladesh team, following General Data Protection Regulation (GDPR) guidelines. The project team in Swansea University who collated the data were all trained in GDPR. The only identifiable participant-level information was the signed consent form for the survey which remained in Bangladesh. The paper questionnaires completed in Bangladesh were stored in a locked cabinet owned by one of the Dhaka site Principal Investigators. The consent forms were held separately to the survey data in a different locked cabinet.

For peer review only

For peer review only - http://bmjopen.bmj.com/site/about/guidelines.xhtml

For peer review only

For peer review only - http://bmjopen.bmj.com/site/about/guidelines.xhtml

References

1. Bangladesh Population Available from: https://worldpopulationreview.com/countries/bangladesh-population.
2. Ahmed SM, Alam BB, Anwar I, Begum T, Huque R, Khan JAM, et al. Bangladesh Health System Review. Pacific ROftW; 2015.
3. Islam MT, Talukder AK, Siddiqui MN, Islam T. Tackling the COVID-19 pandemic: The Bangladesh perspective. J Public Health Res. 2020;9(4):1794. Epub 20201014. doi: 10.4081/jphr.2020.1794. PubMed PMID: 33117758; PubMed Central PMCID: PMCPMC7582102.
4. Asian Development Bank. Poverty Data: Bangladesh. Available from: https://www.adb.org/where-we-work/bangladesh/poverty (Access date 27 November 2023) 2023.
5. Sohrabi C, Alsafi Z, O'Neill N, Khan M, Kerwan A, Al-Jabir A, et al. World Health Organization declares global emergency: A review of the 2019 novel coronavirus (COVID-19). Int J Surg. 2020;76:71-6. Epub 20200226. doi: 10.1016/j.ijsu.2020.02.034. PubMed PMID: 32112977; PubMed Central PMCID: PMCPMC7105032.
6. Bong CL, Brasher C, Chikumba E, McDougall R, Mellin-Olsen J, Enright A. The COVID-19 Pandemic: Effects on Low- and Middle-Income Countries. Anesth Analg. 2020;131(1):86-92. doi: 10.1213/ANE.0000000000004846. PubMed PMID: 32243287; PubMed Central PMCID: PMCPMC7173081.
7. Pasquale S, Gregorio GL, Caterina A, Francesco C, Beatrice PM, Vincenzo P, et al. COVID-19 in Low- and Middle-Income Countries (LMICs): A Narrative Review from Prevention to Vaccination Strategy. Vaccines (Basel). 2021;9(12). Epub 20211214. doi: 10.3390/vaccines9121477. PubMed PMID: 34960223; PubMed Central PMCID: PMCPMC8704834.
8. H.J. Freudenberger HL. Staff burn-out. 30. 1974;1:159-65.
9. World Health Organisation. Burn-out an "occupational phenomenon": International Classification of Diseases. Available from: https://www.who.int/news/item/28-05-2019-burn-out-an-occupational-phenomenon-international-classification-of-diseases (Access date 27 November 2023) 2019.
10. Saladino V, Auriemma V, Campinoti V. Healthcare Professionals, Post-traumatic Stress Disorder, and COVID-19: A Review of the Literature. Front Psychiatry. 2021;12:795221. Epub 20220121. doi: 10.3389/fpsyt.2021.795221. PubMed PMID: 35126205; PubMed Central PMCID: PMCPMC8813735.
11. Buran F, Altin Z. Burnout among physicians working in a pandemic hospital during the COVID-19 pandemic. Leg Med (Tokyo). 2021;51:101881. Epub 2021/04/16. doi: 10.1016/j.legalmed.2021.101881. PubMed PMID: 33857752; PubMed Central PMCID: PMCPMC8025601.
12. AlJhani S, AlHarbi H, AlJameli S, Hameed L, AlAql K, Alsulaimi M. Burnout and coping among healthcare providers working in Saudi Arabia during the COVID-19 pandemic. Middle East Current Psychiatry. 2021;28(29). doi: https://doi.org/10.1186/s43045-021-00108-6.
13. Sasangohar F, Jones SL, Masud FN, Vahidy FS, Kash BA. Provider Burnout and Fatigue During the COVID-19 Pandemic: Lessons Learned From a High-Volume Intensive Care Unit. Anesth Analg. 2020;131(1):106-11. Epub 2020/04/14. doi: 10.1213/ANE.0000000000004866. PubMed PMID: 32282389; PubMed Central PMCID: PMCPMC7173087.
14. Dillon EC, Stults CD, Deng S, Martinez M, Szwerinski N, Koenig PT, et al. Women, Younger Clinicians', and Caregivers' Experiences of Burnout and Well-being During COVID-19 in a US Healthcare System. J Gen Intern Med. 2022;37(1):145-53. Epub 2021/11/04. doi: 10.1007/s11606-021-07134-4. PubMed PMID: 34729697; PubMed Central PMCID: PMCPMC8562379.
15. Khan N, Palepu A, Dodek P, Salmon A, Leitch H, Ruzycki S, et al. Cross-sectional survey on physician burnout during the COVID-19 pandemic in Vancouver, Canada: the role of gender, ethnicity and sexual orientation. BMJ Open. 2021;11(5):e050380. Epub 2021/05/12. doi:

For peer review only - http://bmjopen.bmj.com/site/about/guidelines.xhtml

10.1136/bmjopen-2021-050380. PubMed PMID: 33972345; PubMed Central PMCID: PMCPMC8111871.
16. Cotel A, Golu F, Pantea Stoian A, Dimitriu M, Socea B, Cirstoveanu C, et al. Predictors of Burnout in Healthcare Workers during the COVID-19 Pandemic. Healthcare (Basel). 2021;9(3). Epub 2021/04/04. doi: 10.3390/healthcare9030304. PubMed PMID: 33803286; PubMed Central PMCID: PMCPMC8001536.
17. Duarte I, Teixeira A, Castro L, Marina S, Ribeiro C, Jacome C, et al. Burnout among Portuguese healthcare workers during the COVID-19 pandemic. BMC Public Health. 2020;20(1):1885. Epub 2020/12/09. doi: 10.1186/s12889-020-09980-z. PubMed PMID: 33287794; PubMed Central PMCID: PMCPMC7720923.
18. Greene T, Harju-Seppänen J, Adeniji M, Steel C, Grey N, Brewin CR, et al. Predictors and rates of PTSD, depression and anxiety in UK frontline health and social care workers during COVID-19. European Journal of Psychotraumatology. 2021;12(1):1882781. doi: 10.1080/20008198.2021.1882781.
19. Ahmed T, Musarrat P, Kabir ZN. Lessons learned from pandemic response to COVID-19 in Bangladesh: NGO-based emergency response framework for low- and middle-income countries. BMC Health Serv Res. 2023;23(1):656. Epub 20230620. doi: 10.1186/s12913-023-09643-w. PubMed PMID: 37340495; PubMed Central PMCID: PMCPMC10283326.
20. Hossain MI, Mehedi N, Ahmad I, Ali I, Azman A. Psychosocial stress and trauma during the COVID-19 pandemic: Evidence from Bangladesh. Asian Soc Work Policy Rev. 2021;15(2):145-59. Epub 20210412. doi: 10.1111/aswp.12227. PubMed PMID: 35655964; PubMed Central PMCID: PMCPMC8250397.
21. Talukder B, vanLoon GW, Hipel KW, Orbinski J. COVID-19's implications on agri-food systems and human health in Bangladesh. Curr Res Environ Sustain. 2021;3:100033. Epub 20210310. doi: 10.1016/j.crsust.2021.100033. PubMed PMID: 34977606; PubMed Central PMCID: PMCPMC7945872.
22. Saifuzzaman M, Rahman MM, Shetu SF, Moon NN. COVID-19 and Bangladesh: Situation report, comparative analysis, and case study. Current Research in Behavioral Sciences 2021;2.
23. Siam MHB, Hasan MM, Tashrif SM, Rahaman Khan MH, Raheem E, Hossain MS. Insights into the first seven-months of COVID-19 pandemic in Bangladesh: lessons learned from a high-risk country. Heliyon. 2021;7(6):e07385. Epub 20210625. doi: 10.1016/j.heliyon.2021.e07385. PubMed PMID: 34189333; PubMed Central PMCID: PMCPMC8226055.
24. Yasir Arafat SM, Kabir R. Burnout in Physicians: Global and Bangladesh Perspective. EC Psychology and Psychiatry. 2017;2.3:112-7.
25. Chowdhury SR, Kabir H, Mazumder S, Akter N, Chowdhury MR, Hossain A. Workplace violence, bullying, burnout, job satisfaction and their correlation with depression among Bangladeshi nurses: A cross-sectional survey during the COVID-19 pandemic. PLoS One. 2022;17(9):e0274965. Epub 20220922. doi: 10.1371/journal.pone.0274965. PubMed PMID: 36137141; PubMed Central PMCID: PMCPMC9499253.
26. Biswas M, Hasan MT, Samir N, Alin SI, Homaira N, Hassan MZ, et al. The Prevalence and Associated Factors of Depressive Symptoms Among Medical Students in Bangladesh During the COVID-19 Pandemic: A Cross-Sectional Pilot Study. Front Public Health. 2021;9:811345. Epub 20220131. doi: 10.3389/fpubh.2021.811345. PubMed PMID: 35174136; PubMed Central PMCID: PMCPMC8841836.
27. Zigmond AS, Snaith RP. The hospital anxiety and depression scale. Acta Psychiatr Scand. 1983;67(6):361-70. PubMed PMID: 6880820.
28. Maslach C, Jackson SE. The measurement of experienced burnout. Journal of Occupational Behaviour. 1981;2:99-113.
29. Kalliath TJ, O'Driscoll MP, Gillespie DF, Bluedorn AC. A test of the Maslach burnout inventory in three samples of healthcare professionals Work Stress. 14(1):35-50.

For peer review only - http://bmjopen.bmj.com/site/about/guidelines.xhtml

30. Brady KJS, Ni P, Sheldrick RC, Trockel MT, Shanafelt TD, Rowe SG, et al. Describing the emotional exhaustion, depersonalization, and low personal accomplishment symptoms associated with Maslach Burnout Inventory subscale scores in US physicians: an item response theory analysis. J Patient Rep Outcomes. 2020;4(1):42. Epub 20200601. doi: 10.1186/s41687-020-00204-x. PubMed PMID: 32488344; PubMed Central PMCID: PMCPMC7266903.
31. Maslach C, Jackson SE, Leiter MP. Maslach burnout inventory manual. Menlo Park, CA: Mind Garden Inc, 1996-2008. Report No.
32. Stern AF. The hospital anxiety and depression scale. Occup Med (Lond). 2014;64(5):393-4. doi: 10.1093/occmed/kqu024. PubMed PMID: 25005549.
33. Aly HM, Nemr NA, Kishk RM, Elsaid N. Stress, anxiety and depression among healthcare workers facing COVID-19 pandemic in Egypt: a cross-sectional online-based study. BMJ Open. 2021;11(4):e045281. Epub 2021/05/02. doi: 10.1136/bmjopen-2020-045281. PubMed PMID: 33931409; PubMed Central PMCID: PMCPMC8098284.
34. Ali Sahebi A, Nejati-Zarnaqi B, Moayedi S, Yousefi K, Torres M, Golitaleb M. The prevalence of anxiety and depression among healthcare workers during the COVID-19 pandemic: An umbrella review of meta-analyses. Progress in Neuropsychopharmacology and Biological Psychiatry. 2021;107(110247).
35. Fernandez R, Sikhosana N, Green H, Halcomb EJ, Middleton R, Alananzeh I, et al. Anxiety and depression among healthcare workers during the COVID-19 pandemic: a systematic umbrella review of the global evidence. BMJ Open. 2021;11(9):e054528. Epub 2021/09/23. doi: 10.1136/bmjopen-2021-054528. PubMed PMID: 34548373; PubMed Central PMCID: PMCPMC8458002.
36. Yin C, Ji J, Cao X, Jin H, Ma Q, Gao Y. Impact of long working hours on depressive symptoms among COVID-19 frontline medical staff: The mediation of job burnout and the moderation of family and organizational support. Front Psychol. 2023;14:1084329. Epub 20230215. doi: 10.3389/fpsyg.2023.1084329. PubMed PMID: 36874813; PubMed Central PMCID: PMCPMC9975257.
37. Bari R, Sultana F. Second Wave of COVID-19 in Bangladesh: An Integrated and Coordinated Set of Actions Is Crucial to Tackle Current Upsurge of Cases and Deaths. Front Public Health. 2021;9:699918. Epub 20210830. doi: 10.3389/fpubh.2021.699918. PubMed PMID: 34527649; PubMed Central PMCID: PMCPMC8437241.

For peer review only

For peer review only - http://bmjopen.bmj.com/site/about/guidelines.xhtml

Figures

Figure 1. HADS Anxiety and Depression scores by institution.

Figure 2. MBI scores for EE, DP and PA by institution.

For peer review only

For peer review only - http://bmjopen.bmj.com/site/about/guidelines.xhtml

Tables

For peer review only

For peer review only - http://bmjopen.bmj.com/site/about/guidelines.xhtml

Table 1: Demographic information by Institution

	Institution			
	**SRNGIH n=400**	**DMCH n=1283**	**MMCH n=628**	**MARMC n=394**
**Age (years)**				
Mean (s.d.)	32.2 (7.3)	33.6 (8.4)	37.1 (8.1)	32.3 (7.4)
**Gender (%)**				
Female: Male	72:28	82:18	71:29	83:17
**Departments (n,%)**				
Internal Medicine	12 (3)	227 (17.7)	99 (15.7)	57 (14.5)
Cardiology	1 (0.3)	1 (0.1)	12 (1.9)	26 (6.6)
Gastroenterology	207 (51.8)	6 (0.5)	9 (1.4)	0
Respiratory Medicine	4 (1.0)	7 (0.6)	8 (1.3)	0
Nephrology	1 (0.3)	55 (4.3)	20 (3.2)	16 (4.1)
Neurology	0	25 (1.9)	2 (0.3)	9 (2.3)
Rheumatology	3 (0.8)	3 (0.2)	5(0.8)	1 (0.3)
Surgery	21 (5.3)	280 (21.8)	69 (11.0)	83 (21.1)
Gynae/Obs	1 (0.3)	124 (9.7)	57 (9.1)	38 (9.6)
Psychiatry	1 (0.3)	8 (0.6)	4 (0.6)	0
Other	149 (37.3)	533 (41.5)	340 (54.1)	156 (39.6)
	0	14 (1.1)	3 (0.5)	8 (2.0)
**Professions (n,%)**				
Academic	31 (7.8)	2 (0.2)	119 (18.9)	5 (1.3)
Doctor	89 (22.3)	157 (12.2)	193 (30.7)	76 (19.3)
Nurse	277 (69.3)	1092 (85.1)	311 (49.5)	312 (79.2)
Other	3 (0.8)	30 (2.3)	4 (0.6)	1 (0.3)
Nmiss (%)	0	2 (0.2)	1 (0.2)	0
**COVID rota (n,%)**				
Yes	357 (89.3)	490 (38.2)	609 (97.0)	201 (51.0)
No	43 (10.8)	792 (61.7)	19 (3.0)	193 (49.0)
**Govt. Employee**				
Yes	395 (98.8)	1146 (89.3)	624 (99.4)	386 (98.0)
No	5 (1.3)	137 (10.7)	3 (0.5)	8 (2.0)
nmiss(%)	0	0	1 (0.2)	0
**Private Practice**				
Yes	76 (19.0)	29 (2.3)	117 (18.6)	8 (2.0)
No	324 (81.0)	1252 (97.6)	510 (81.2)	86 (98.0)
**Wellbeing Service**				
Yes	190 (47.5)	606 (47.2)	244 (38.9)	174 (44.2)
No	209 (52.3)	672 (52.4)	383 (60.9)	214 (54.3)
**Wellbeing Service (Getting help) [multiple response]**				
Friends/Family	20 (10.5)	63 (10.4)	48 (19.7)	4 (2.3)
Work Colleagues	93 (49.0)	356 (58.8)	110 (45.1)	120 (69.0)
Occupational Health	10 (5.3)	15 (2.5)	5 (2.1)	14 (8.1)
None	66 (34.7)	172 (28.4)	84 (34.4)	38 (21.8)
Other	2 (1.1)	9 (1.5)	0	0

For peer review only

Table 2: Hospital Anxiety and Depression Score (HADs) and Maslach Burnout Scores by Institution

	Institutions					
**HADs**	**SRNGIH**	**DMCH**	**MMCH**	**MARMC**	**F-test (*p-value*)**	**Tukey *t-test (significant)*[1]**
**Anxiety Subscale Score** Mean (s.d.)	7.6 (4)	7.8 (4)	8.6 (4.1)	9 (4.3)	14.7 (<0.01)	G1 vs G2<br>G1 vs G3*<br>G1 vs G4*<br>G2 vs G3*<br>G2 vs G4*<br>G3 vs G4
**Depression Subscale Score** Mean (s.d.)	6.3 (4.1)	6.6 (3.7)	7.9 (3.9)	6.9 (4.5)	17.3 (<0.01)	G1 vs G2<br>G1 vs G3*<br>G1 vs G4<br>G2 vs G3*<br>G2 vs G4<br>G3 vs G4*
**Maslach Subscale Scores**	**SRNGIH**	**DMCH**	**MMCH**	**MARMC**	**F-test (*p-value*)**	**Tukey *t-test (significant)*[1]**
**Emotional exhaustion (EE)** Mean (s.d.)	17.5 (11.1)	15.4 (10.0)	19.1 (10.8)	15.7 (8.1)	20.6 (*p<0.01*)	G1 vs G2*<br>G1 vs G3<br>G1 vs G4<br>G2 vs G3*<br>G2 vs G4<br>G3 vs G4*
**Depersonalisation / loss of empathy (DP)** Mean (s.d.)	4.3 (5.3)	4.3 (5.1)	5.2 (5.2)	4.7 (5.8)	4.11 (*p<0.01*)	G1 vs G2<br>G1 vs G3*<br>G1 vs G4<br>G2 vs G3*<br>G2 vs G4<br>G3 vs G4
**Personal accomplishment assessment (PA)** Mean (s.d.)	39.0 (8.4)	38.5 (8.8)	36.5 (9.4)	37.8 (7.4)	8.9 (*p<0.01*)	G1 vs G2<br>G1 vs G3*<br>G1 vs G4<br>G2 vs G3*<br>G2 vs G4<br>G3 vs G4

For peer review only - http://bmjopen.bmj.com/site/about/guidelines.xhtml

Table 3: Hospital Anxiety and Depression Score (HADs) and Maslach Burnout Scores by COVID duty

	COVID Duty		*t*-test (*p*-value)
**HADs**	**Yes**	**No**	
**Anxiety Subscale Score** Mean (s.d.)	8.3 (4.2)	7.8 (3.9)	3.4 (*p<0.01*)
**Depression Subscale Score** Mean (s.d.)	7.1 (4.2)	6.5 (3.7)	4.2 (p<0.01)
**Maslach Subscale Scores**	**Yes**	**No**	**Chi-Square (*p-value*)**
**Emotional exhaustion (OE)** Mean (s.d.)	17.5 (10.4)	15.3 (9.8)	5.4 (p<0.01)
**Depersonalisation / loss of empathy (DP)** Mean (s.d.)	4.9 (5.4)	4.1 (5.0)	3.9 (p<0.01)
**Personal accomplishment assessment (PA)** Mean (s.d.)	37.3 (9.1)	39.2 (8.1)	-5.4 (p<0.01)

For peer review only

For peer review only - http://bmjopen.bmj.com/site/about/guidelines.xhtml

Figure 1. HADS Anxiety and Depression scores by institution.

a) HADs Anxiety scores*

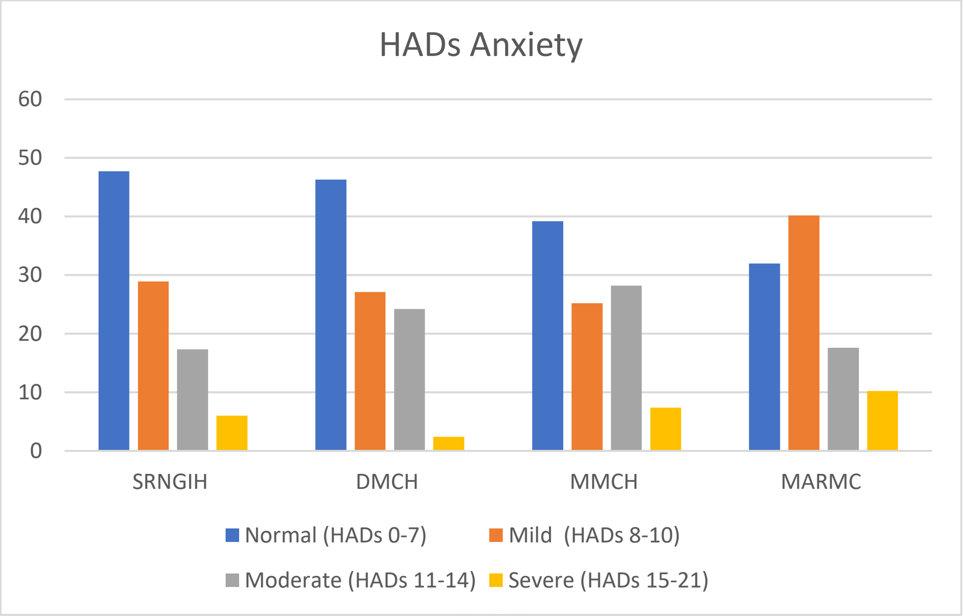

b) HADs Depression scores*

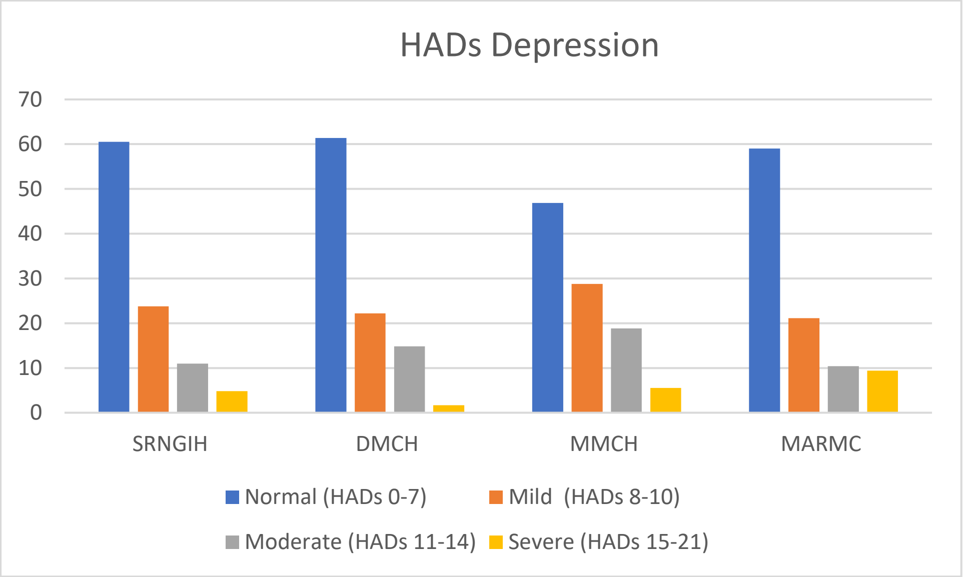

Figures indicate the proportion of participants in each institution with normal (0-7), mild (8-10) moderate (11-14) or severe (15-21): a) HADs Anxiety and b) HADs Depression scores (*indicates a statistically significant difference in proportions, p<0.05, across institutions).

Figure 2. MBI scores for EE, DP and PA by institution.

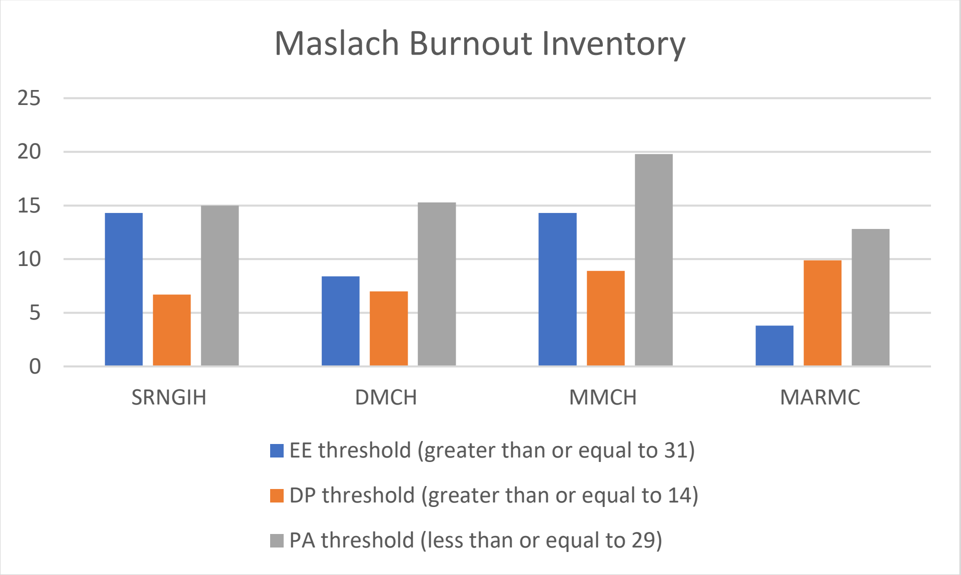

Maslach Burnout Inventory (MBI) %	SRNGIH	DMCH	MMCH	MARMC
EE threshold (greater than or equal to 31)*	14.3	8.4	14.3	3.8
DP threshold (greater than or equal to 14)	6.7	7	8.9	9.9
PA threshold (less than or equal to 29)*	15	15.3	19.8	12.8

Proportion of participants in each institution achieving threshold levels for burnout (EE-greater than or equal to 31; DP- greater than or equal to 14; PA- less than or equal to 29) (*indicates a statistically significant difference in proportions, p<0.05, across institutions).

For peer review only

Supplementary information Table S1: STROBE checklist for crosse-sectional studies indicating where relevant information is found in the manuscript.

Section	Item no	Recommendation	Page no in manuscript
**Title and abstract**	1	*(a)* Indicate the study's design with a commonly used term in the title or the abstract<br>*(b)* Provide in the abstract an informative and balanced summary of what was done and what was found	1<br>2-3
**Introduction**			
Background/rationale	2	Explain the scientific background and rationale for the investigation being reported	6
Objectives	3	State specific objectives, including any prespecified hypotheses	6
**Methods**			
Study design	4	Present key elements of study design early in the paper	7
Setting	5	Describe the setting, locations, and relevant dates, including periods of recruitment, exposure, follow-up, and data collection	7
Participants	6	(*a*) Give the eligibility criteria, and the sources and methods of selection of participants	7
Variables	7	Clearly define all outcomes, exposures, predictors, potential confounders, and effect modifiers. Give diagnostic criteria, if applicable	7, 8
Data sources/ measurement	8*	For each variable of interest, give sources of data and details of methods of assessment (measurement). Describe comparability of assessment methods if there is more than one group	7, 8
Bias	9	Describe any efforts to address potential sources of bias	8
Study size	10	Explain how the study size was arrived at	7
Quantitative variables	11	Explain how quantitative variables were handled in the analyses. If applicable, describe which groupings were chosen and why	9,10
Statistical methods	12	*(a)* Describe all statistical methods, including those used to control for confounding<br>*(b)* Describe any methods used to examine subgroups and interactions<br>*(c)* Explain how missing data were addressed<br>*(d)* If applicable, describe analytical methods taking account of sampling strategy<br>*(e)* Describe any sensitivity analyses	7,8, 9,10
**Results**			
Participants	13*	(a) Report numbers of individuals at each stage of study—eg numbers potentially eligible, examined for eligibility, confirmed eligible, included in the study, completing follow-up, and analysed<br>(b) Give reasons for non-participation at each stage<br>(c) Consider use of a flow diagram	10
Descriptive data	14*	(a) Give characteristics of study participants (eg demographic, clinical, social) and information on exposures and potential confounders	10, 11, 12

For peer review only

		(b) Indicate number of participants with missing data for each variable of interest	Table 1, 24
Outcome data	15*	Report numbers of outcome events or summary measures	10, 11, 12<br>23, 24, 25<br>Tables 1, 2, 3
Main results	16	*(a)* Give unadjusted estimates and, if applicable, confounder-adjusted estimates and their precision (eg, 95% confidence interval). Make clear which confounders were adjusted for and why they were included<br>*(b)* Report category boundaries when continuous variables were categorized<br>*(c)* If relevant, consider translating estimates of relative risk into absolute risk for a meaningful time period	10, 11, 12<br>23, 24, 25<br>Tables 1, 2, 3
Other analyses	17	Report other analyses done—eg analyses of subgroups and interactions, and sensitivity analyses	N/A
**Discussion**			
Key results	18	Summarise key results with reference to study objectives	13
Limitations	19	Discuss limitations of the study, taking into account sources of potential bias or imprecision. Discuss both direction and magnitude of any potential bias	14
Interpretation	20	Give a cautious overall interpretation of results considering objectives, limitations, multiplicity of analyses, results from similar studies, and other relevant evidence	13, 14, 15
Generalisability	21	Discuss the generalisability (external validity) of the study results	13, 14, 15
**Other information**			
Funding	22	Give the source of funding and the role of the funders for the present study and, if applicable, for the original study on which the present article is based	15

*Give information separately for exposed and unexposed groups.

For peer review only

BMJ Open

Did the COVID-19 pandemic affect levels of burnout, anxiety and depression among doctors and nurses in Bangladesh?: A cross-sectional survey study

Journal:	*BMJ Open*
Manuscript ID	bmjopen-2023-079350.R2
Article Type:	Original research
Date Submitted by the Author:	18-Jan-2024
Complete List of Authors:	Hutchings, Hayley; Swansea University<br>Rahman, Mesbah; Swansea Bay University Health Board<br>Carter, Kymberley; Swansea University<br>Islam, Saiful; Swansea University<br>O'Neill, Claire; Swansea University<br>Roberts, Stephen; Swansea University<br>John, Ann; Swansea University<br>Fegan, Greg; Swansea University<br>Dave, Umakant; Swansea Bay University Health Board<br>Hawkes, Neil; Cwm Taf Morgannwg University Health Board<br>Ahmed, Faruque; Sheikh Russel National Gastroliver Institute and Hospital, Department of Gastroenterology<br>Hasan, Mahmud; Gastroliver Foundation<br>Azad, Abul Kalam; Dhaka Medical College and Hospital<br>Rahman, Md.; Dhaka Medical College and Hospital<br>Golam Kibria, Md; Sheikh Russel National Gastroliver Institute<br>Rahman, M. Masudur ; Sheik Russel National Gastroliver Institute<br>Mia, Titu; Mugda Medical College and Hospital<br>Akhter, Mahfuza; Mugda Medical College and Hospital<br>Williams, John; Swansea University, Faculty of Medicine, Health and Life Science
**Primary Subject Heading**:	Global health
Secondary Subject Heading:	Health services research, Mental health
Keywords:	COVID-19, Anxiety disorders < PSYCHIATRY, Depression & mood disorders < PSYCHIATRY, Burnout, Professional

SCHOLARONE™
Manuscripts

I, the Submitting Author has the right to grant and does grant on behalf of all authors of the Work (as defined in the below author licence), an exclusive licence and/or a non-exclusive licence for contributions from authors who are: i) UK Crown employees; ii) where BMJ has agreed a CC-BY licence shall apply, and/or iii) in accordance with the terms applicable for US Federal Government officers or employees acting as part of their official duties; on a worldwide, perpetual, irrevocable, royalty-free basis to BMJ Publishing Group Ltd ("BMJ") its licensees and where the relevant Journal is co-owned by BMJ to the co-owners of the Journal, to publish the Work in this journal and any other BMJ products and to exploit all rights, as set out in our licence.

The Submitting Author accepts and understands that any supply made under these terms is made by BMJ to the Submitting Author unless you are acting as an employee on behalf of your employer or a postgraduate student of an affiliated institution which is paying any applicable article publishing charge ("APC") for Open Access articles. Where the Submitting Author wishes to make the Work available on an Open Access basis (and intends to pay the relevant APC), the terms of reuse of such Open Access shall be governed by a Creative Commons licence – details of these licences and which Creative Commons licence will apply to this Work are set out in our licence referred to above.

Other than as permitted in any relevant BMJ Author's Self Archiving Policies, I confirm this Work has not been accepted for publication elsewhere, is not being considered for publication elsewhere and does not duplicate material already published. I confirm all authors consent to publication of this Work and authorise the granting of this licence.

For peer review only

Did the COVID-19 pandemic affect levels of burnout, anxiety and depression among doctors and nurses in Bangladesh?: A cross-sectional survey study

Hayley A Hutchings[1]*, Mesbah Rahman[2], Kymberley Carter[1], Saiful Islam[1], Claire O'Neill[1], Stephen Roberts[1], Ann John[1], Greg Fegan[1], Umakant Dave[2], Neil Hawkes[3], Faruque Ahmed[4], Mahmud Hasan[5], Abul Kalam Azad[6], Mujibur Rahman[6], Md Golam Kibria[7], Masudur Rahman[7], Titu Mia[8], Mahfuza Akhter[8], John G Williams

Author Affiliations

[1]School of Medicine, Faculty of Medicine, Health and Life Science, Swansea University, Swansea, United Kingdom.

[2]Swansea Bay University Health Board, Swansea, United Kingdom.

[3]Cwm Taf Morgannwg University Health Board, Abercynon, United Kingdom.

[4] Department of Gastroenterology, Sheikh Russel National Gastroliver Institute and Hospital, Dhaka, Bangladesh.

[5] Gastroliver Foundation, Dhaka, Bangladesh.

[6]Dhaka Medical College and Hospital, Dhaka, Bangladesh.

[7]Sheikh Russel National Gastroliver Institute, Dhaka, Bangladesh.

[8]Mugda Medical College and Hospital, Dhaka, Bangladesh.

*Corresponding author

Email: h.a.hutchings@swansea.ac.uk

For peer review only - http://bmjopen.bmj.com/site/about/guidelines.xhtml

Abstract

Introduction

COVID-19 has caused severe disruption to clinical services in Bangladesh but the extent of this, and the impact on healthcare professionals is unclear. We aimed to assess the perceived levels of anxiety, depression and burnout among doctors and nurses during COVID-19 pandemic.

Methods

We undertook an online survey using RedCap, directed at doctors and nurses across four institutions in Bangladesh (The Sheikh Russel Gastro Liver Institute & Hospital (SRNGIH), Dhaka Medical College Hospital (DMCH), Mugda Medical College Hospital (MMCH), and M Abdur Rahim Medical College (MARMC) hospital. We collected information on demographics, awareness of wellbeing services, COVID-19 -related workload, as well as anxiety, depression and burnout using two validated questionnaires: the Hospital Anxiety and Depression Scale (HADS); and the Maslach Burnout Inventory (MBI).

Results

Of the 3000 participants approached, we received responses from 2705 (90.2%). There was a statistically significant difference in anxiety, depression and burnout scores across institutions (P< 0.01). Anxiety, depression and burnout scores were statistically worse in COVID-19 active staff compared with those not working on COVID-19 activities (P< 0.01 for HADS anxiety and depression and MBI EE, DP and PA). Over half of the participants exhibited some level of anxiety (SRNGIH- 52.2%; DMCH- 53.9%; MMCH- 61.3%; MARMC- 68%) with a high proportion experiencing depression (SRNGIH- 39.5%; DMCH- 38.7%; MMCH- 53.7%; MARMC- 41.1%). Although mean burnout scores were within the normal range for each

For peer review only - http://bmjopen.bmj.com/site/about/guidelines.xhtml

institution, a high proportion of staff (almost 20% in some instances) were shown to be classified as experiencing burnout by their EE, DP, and PA scores.

Conclusion

We identified a high prevalence of perceived anxiety, depression, and burnout among doctors and nurses during the COVID-19 pandemic. This was worse in staff engaged in COVID-19 related activities. These findings could help healthcare organisations to help plan for future similar events.

297 words

For peer review only - http://bmjopen.bmj.com/site/about/guidelines.xhtml

Word count- 2664

Key words

COVID-19; health personnel; anxiety; depression; burnout, professional

Strengths and Limitations of the study

- We conducted a large-scale cross-sectional survey using RedCap™, across four institutions in Bangladesh with nurses and doctors.
- We employed two validated measures to assess anxiety, depression and burnout.
- The study was operationalised by personnel based in Bangladesh, whilst the analysis was conducted remotely in the U.K.
- Further work is needed to determine whether anxiety, depression and burnout persist post COVID-19.
- We did not explore whether differences in staff characteristics and awareness of wellbeing services across institutions had any effect on levels of anxiety, depression and burnout.

For peer review only

Introduction

Bangladesh is classified as a Low and/or Middle-Income Country (LMIC). It has a population of171 million people and a population density of 1,115.62 people per square kilometre, which ranks 10th in the world[1]. The health system of Bangladesh is a pluralistic system with four key sectors: government, private sector, nongovernmental organizations (NGOs) and donor agencies[2]. Bangladesh's economy has been growing with a GDP growth rate of more than 7.5%; however, a high proportion of the population (about 20%) are living below the poverty line[3, 4]. Bangladesh has only 5.3 doctors per 10,000 people, 0.3 nurses per 1,000 people, 0.87 hospital beds per 1,000 people, 0.72 ICU beds and 1.1 ventilators per 100,000 people[3].

On January 30th, 2020, the World Health Organisation (WHO) declared the outbreak of COVID-19 a Public Health Emergency of International Concern[5]. According to the WHO COVID-19 was likely to have the biggest impact in countries with vulnerable health care systems. Due to its high population density, poor health care systems, poverty and a weak economy Bangladesh had the potential to be at risk of poor outcomes from COVID-19[3]. Bangladesh reported their first case of COVID-19 on March 8, 2020[3, 6, 7].

The concept of 'staff burnout' was identified in 1974 by Freudenberger[8]. It was defined as "burnout in the individual's energy due to failure, wear-out, overloading, and unfulfilled desires". In 2019, 'burnout' was recognised by the World Health Organisation (WHO) as an 'occupational phenomenon'[9], with a dedicated entry in its International Classification of Diseases Handbook (ICD-11 for Mortality and Morbidity Statistics (who.int)).

For peer review only - http://bmjopen.bmj.com/site/about/guidelines.xhtml

Since the beginning of the COVID-19 pandemic there has been extensive research exploring the impact of the pandemic on stress, anxiety, depression and burnout within the healthcare community[10]. Researchers in Turkey, Saudi Arabia, United States, Canada, Romania, UK, and Portugal have demonstrated the negative impact that COVID-19 has had on levels of burnout, stress and anxiety[11-18]. Factors such as worries about legal issues, age, experience, longer working hours, risk of exposing family members to COVID-19, direct COVID-19 facing roles, being redeployed, gender and coping strategies were related to levels of burnout and stress.

COVID-19 has caused severe disruption in Bangladesh. This has included the country's finances, the delivery of clinical services and its impact on the people of Bangladesh[19-23]. Research in other countries has documented the impact of COVID-19 on healthcare workers, but most of this has been undertaken in higher income countries[11-18].

The potential impact that burnout could have from the Bangladesh perspective has previously been considered. Factors including time, pressure, workload and family issues have been identified as being important.[24]. Research exploring work stresses in nurses identified that depression was positively associated with workplace violence, bullying and burnout[25]. Female gender, increased social media use, low levels of optimism and sleeping problems increased the risk of depression in trainee medical students during the pandemic in Bangladesh[26]. There has however been limited large-scale work exploring the impact of COVID-19 on anxiety, depression and burnout in healthcare workers in Bangladesh. The aim of this study was therefore to explore levels of burnout, anxiety and depression in doctors and nurses within Bangladesh during the COVID-19 pandemic. We hypothesised that those

For peer review only - http://bmjopen.bmj.com/site/about/guidelines.xhtml

staff involved in COVID-19 frontline activities would have worse burnout, anxiety and depression than those staff not engaged in COVID-19 frontline activities.

Materials and Methods

Study sites

A cross-sectional survey study was initiated between June and August 2021 in four large institutions within Bangladesh. We hadthree sites in Dhaka and one in Dinajpur (northern Bangladesh): The Sheikh Russel Gastro Liver Institute & Hospital (SRNGIH), Dhaka Medical College Hospital (DMCH), Mugda Medical College Hospital (MMCH), and M Abdur Rahim Medical College (MARMC) hospital.

Study survey

The survey sought information on personal wellbeing and current awareness of local wellbeing services from doctors and nurses working across all specialties at the four institutions. The total number of healthcare staff across the four institutions was 3000 at the time of the survey. The study team included a local clinician at each of the four sites who acted as the site principal investigator (PI). Each PI sent an email on behalf of the study team to all clinical staff at their institution inviting them to participate.

We used validated questionnaires to measure stress and anxiety. We used the Hospital Anxiety and Depression Scale-HADS[27] to measure Anxiety and Depression. The HADS has been previously demonstrated to have good reliability, with cronbach's alpha values between 0.68 and 0.93 for the anxiety and 0.67 and 0.9 for the depression sub-scales respectively[28].

For peer review only - http://bmjopen.bmj.com/site/about/guidelines.xhtml

A higher score on the HADS indicates a worse anxiety/depression level. In terms of score interpretation, a HADS score of 0-7 is regarded as normal, 8-10 mild, 11-14 moderate and 15-21 severe[27].

We used the Maslach Burnout Inventory- MBI[29] for Healthcare staff[30] to measure burnout in the form of: Emotional Exhaustion (EE), Depersonalisation/loss of empathy (DP), and Personal Accomplishment (PA). Reliability of the MBI in healthcare staff has been demonstrated to be acceptable (>0.7 for all sub-scales)[31, 32]. Higher scores on the EE and DP subscales of the MBI indicate a higher burnout symptom burden; lower scores on the PA subscale indicate a higher burnout symptom burden[33]. We used previously published MBI raw score cut-points to assess burnout: ≥ 31, ≥ 14, and ≤ 29 respectively for the EE, DP, and PA subscales[33].

In addition to the two validated questionnaires, we asked participants at which institution they were based, their job/profession, their gender, their age, whether they were working on a COVID-19 rota, whether they were a government employee, whether they undertook any private work and whether they were aware of any well-being services available to them. We also asked them if they had any difficulties answering any of the questions on the HADS or MBI. We provided participant information sheets and consent forms in Bengali and the HADS and MBI in English. Although there was a validated Bengali version of the HADS, there was no Bengali translation for the MBI. Therefore as most of the participants were English speaking, we opted to use English versions of both questionnaires. We provided additional guidance in Bengali to help participants complete these surveys (if required) and the local PI was available at each institution to provide further guidance, if necessary.

For peer review only - http://bmjopen.bmj.com/site/about/guidelines.xhtml

We invited doctors and nurses working both in frontline COVID-19 roles (i.e those directly managing COVID-19 patients) as well as those not to participate in the survey in order for comparisons to be made. Once participants had consented to take part, we provided the survey either on paper or as a link to an online survey via email. We collated the responses and they were entered onto an electronic data capture system REDCap by the study coordinator in Bangladesh. The REDCap database was hosted in Swansea and data analysis was performed by a Swansea based Bangladeshi statistician.

Public and Patient Involvement

None

Analysis

For the validated surveys we calculated scores and used the standard processes to manage missing data in line with developers recommendations[27, 34, 35]. We explored the completeness of the data from all the surveys and sites and investigated any systematic errors and biases. We looked for evidence of data being Missing Completely At Random (MCAR). In the analysis, the number and percentages of missing data are provided for all the outcomes.

The HADS anxiety and depression scores and the subscales of the MBI (emotional exhaustion (EE), depersonalization (DP), and personal accomplishment (PA)) are presented using summary statistics (mean and standard deviation (s.d.).

For peer review only - http://bmjopen.bmj.com/site/about/guidelines.xhtml

We used one-way ANOVA F-tests to compare anxiety, depression and burnout scores across the different institutions. We used Tukey post hoc tests to identify where significant differences existed between different groups after the ANOVA test. We used unpaired t- tests and chi-squared tests to compare scores from participants that actively worked on COVID-19 frontline activities vs non-COVID-19 frontline activities. A p value of less than 0.05 was regarded as significant. Chi-squared tests were used to determine if there was any difference in the proportion of participants reaching threshold levels for anxiety, burnout and depression between institutions. Post-hoc tests with bonferroni corrections were employed to identify where (if any) the differences existed between institutions. Analysis of the data was carried out using Stata™.

Results

Two thousand seven hundred and five (2705) participants out of 3000 (90.2%) responded to our survey. Of the 2705 responses, we received 400 (14.8%) from SRNGIH, 1283 (47.4%) from DMCH, 628 (23.2%) from MMCH and 394 (14.6%) from MARMC. In total, 2108/2705 (77.9%) of the responses were from females and 592/2705 (21.9%) from males, with 5 (0.2%) responses where gender was not specified. Table 1 illustrates the demographic characteristics across the four institutions.

Table 2 illustrates the HADS scores across the four institutions. There was a statistically significant difference in HADS scores across the different institutions ($p<0.05$). The mean anxiety subscale score was in the normal range (0-7) for two institutions (DMCH and MMCH) and in the mild range (8-10) in the other two institutions. In terms of the mean depression

For peer review only - http://bmjopen.bmj.com/site/about/guidelines.xhtml

subscale score three of the four institutions had depression scores within the normal range (SRNGIH, DMCH and MARMC), with the fourth institution (MMCH) having a mean score just above the normal range. Figure 1 illustrates the proportion of participants at each institution with normal (0-7), mild (8-10), or abnormal (11-14 moderate; 15-21 severe) HADS anxiety and depression scores. Over half of the participants exhibited some level of anxiety (SRNGIH- 52.2%; DMCH- 53.9%; MMCH- 61.3%; MARMC- 68%) with a high proportion experiencing depression (SRNGIH- 39.5%; DMCH- 38.7%; MMCH- 53.7%; MARMC- 41.1%). There was a statistically significant difference in the proportion of participants experiencing anxiety or depression across the different institutions (P<0.05).

Table 2 illustrates the Maslach scores across the four institutions. There was a statistically significant difference in scores across the four institutions (p<0.05). Mean EE, DP and PA scores did not indicate burnout. Figure 2 illustrates the proportion of staff at each institution who had scores that were classified as reaching the cut-off points that are classified as defining burnout for EE, DP, and PA scores[33]. Therefore, although mean scores were within the normal range for each institution, a high proportion of staff (almost 20% in some instances) were shown to be classified as experiencing burnout by their Maslach EE, DP and PA scores. There was a statistically significant difference (p<0.05) in the proportion of participants by institution experiencing burnout as indicated by their EE and PA scores. There was however no significant difference in scores across institutions by DP scores (p>0.05).

As we expected the institution with the highest proportion of COVID-19 rota working (MMCH- 97%) had the highest HADS anxiety and depression scores. MMCH also had the highest MBI scores for the EE and DP subscales of all the institutions and the lowest MBI scores for PA,

For peer review only - http://bmjopen.bmj.com/site/about/guidelines.xhtml

indicating higher levels of burnout as well as the most individuals achieving these burnout scores.

Table 3 illustrates the impact of a COVID-19 frontline role on HADS and Maslach scores. Healthcare professionals who were engaged with frontline COVID-19 duties had a statistically significantly higher anxiety (8.3 vs 7.8; $p<0.01$) and depression (7.1 vs 6.5; $p<0.05$) scores when compared with healthcare professionals who were not engaged with COVID frontline duties. Similarly, Maslach scores for mean EE scores (17.5 vs 15.3; $p<0.05$) and DP scores (4.9 vs 4.1; $p<0.01$) were statistically significantly higher in staff with frontline COVID-19 duties compared with staff not in frontline COVID-19 duties, indicating more burnout. Scores for Maslach PA were lower (37.3 vs 39.2; $p<0.01$) in COVID-19 duty staff than non-COVID-19 duty staff, again indicating worse burnout.

For peer review only - http://bmjopen.bmj.com/site/about/guidelines.xhtml

Discussion

In this cross-sectional survey we found that a high proportion of staff working in the four hospitals had some level of anxiety (greater than 50% of participants) and depression (almost 40% of participants). Although the mean MBI scores for the EE, DP and PA scales were within the normal range, a high proportion of participants exceeded the thresholds defined as indicating burnout[33]. There were statistically significant differences across the four institutions for the HADS and MBI scores. We also found that those participants working on COVID-19 activities had worse anxiety, depression and burnout scores that participants that were not working on COVID-19 related activities. The differences in scores across institutions could be explained by the numbers of participants in each institution that were COVID-19 active, as MMCH had the worse anxiety, depression and burnout scores and had the highest number of COVID-19 active staff.

The prevalence of stress, anxiety and depression has been measured in previous studies undertaken throughout the pandemic period. Our findings concur with these studies where all have consistently identified a high prevalence of perceived stress, anxiety and depression among healthcare workers.[36, 37]. A systematic umbrella review reported that there were differences in the prevalence of anxiety and depression amongst different healthcare workers, but that the prevalence was at least 17% and up to 40% in some groups[38]. As with our findings, frontline workers appear to be most affected by anxiety and depression[12].

A study in Bangladeshi nurses during the pandemic showed that depression was linked with burnout and that longer working hours contributed to higher depression[25]. The increased

For peer review only - http://bmjopen.bmj.com/site/about/guidelines.xhtml

workload associated with the pandemic has also been shown to result in higher levels of depression and burnout[39], findings that were also mirrored in our study. A recent pilot study in Bangladesh similarly identified a very high prevalence of depression symptoms among Bangladeshi medical students[26].

Our study has several strengths. We had a large survey sample size of more than 2700 participants with an excellent response rate (90%) that surveyed multiple disciplines and different job roles in four major hospitals in Bangladesh. All information were collected anonymously which may have contributed to the willingness of participants to respond. There were some limitations. The survey tools themselves were administered in English as there was no validated Bengali translation for one of the measures, and because most of the staff were English speaking. We did however provide some supplementary information in Bengali to aid completion and added questions at the end of the survey asking for feedback about whether they had understood the survey questions. The responses returned indicated that there were no major problems with comprehension. The high response rate is also likely to indicate that most participants did not experience problems with completion.

The survey itself was limited to doctors and nurses who are likely to have good levels of education. If the survey was rolled out to less qualified staff who may have poorer understanding of the English language, translation into their native language would be recommended. This survey was conducted during the second wave of COVID-19[40] when stresses on healthcare workers were likely to be high. Further work is needed to explore whether the levels of anxiety, depression, and burnout persist in Bangladesh post pandemic.

For peer review only - http://bmjopen.bmj.com/site/about/guidelines.xhtml

For peer review only

A further limitation is that our analysis did not control for covariates, in particular, the characteristics of the participants and their awareness of wellbeing services across institutions. It is possible therefore that the increased levels of anxiety, depression and burnout identified in those more engaged in COVID-19 frontline activities may be due to differences in participant characteristics and/or differences in awareness of well-being services. Future studies should explore the impact (if any) of these factors on levels of anxiety, depression, and burnout.

Our findings suggest that working in the healthcare sector in busy and stressful situations may impact on anxiety, depression, and burnout. However, as we did not have baseline data from before the COVID-19 pandemic for comparison it is impossible to definitively conclude this. The findings do suggest that healthcare workers may have higher rates than the general population of Bangladesh and prior the COVID-19 pandemic[41]. Healthcare staff levels of anxiety, depression and burnout were worse if they had frontline COVID-19 duties.

Healthcare organisations need to consider how to plan for large-scale events such as future pandemics to ensure that staff can be appropriately supported during such times whilst maintaining delivery of high-quality healthcare.

Acknowledgments

Jayne Price for her administrative support on the project, staff from Bangladesh involved in data collection/entry, all healthcare professionals who completed the survey.

For peer review only - http://bmjopen.bmj.com/site/about/guidelines.xhtml

Contributorship Statement

HAH, JGW, CON, KC, GF and Mesbah Rahman (MesR) conceived and secured funding for the study. HAH was the study chief investigator. KC was the research manager and was responsible for overseeing and securing all governance approvals. SI led on data management and statistical analysis with oversight from GF. SR and AJ provided input on study design and interpretation. UD, NH and MesR provided clinical input to the study. FA, MH, AKA, MR, MGK, Masudur Rahman (MasR), TM, MA were the Bangladesh principal investigators and were responsible for leading on institutional ethics approvals, site set-up and local recruitment and data collection. HAH led on the drafting of the manuscript. All reviewed and provided input on the final manuscript. HAH is the guarantor for the manuscript and accepts full responsibility for the work and/or the conduct of the study, had access to the data and controlled the decision to publish.

Competing Interests

None

Funding

This study was funded by Swansea University through their Global Challenges Research Fund.

Grant number: N/A.

Data Sharing Statement

Data are available upon reasonable request.

Ethics approval

The study was approved by the Swansea University Medical School Research Ethics Sub-Committee (Ref: 2020-0070), as well as the Ethics Boards at each of the four institutions (Sheik Russel Gastroliver Institute and Hospital Institutional Ethical Review Committee (Ref: SRG&H/ERC/2020-2021/02); M Abdur Rahim Medical College Institutional Review Board (Ref: MARMCD/2021/370); Mugda Medical College & Hospital Institutional Review Board (no ref); Dhaka Medical College Institutional Review Board (Ref: ERC-DMC-ECC/2021/47)).

We provided participants with an information sheet prior to them participating and all were required to sign a written consent form before proceeding with providing questionnaire responses for the survey. Information about participants was kept confidential and managed in accordance with the Data Protection Act, NHS Caldicott Guardian, The Research Governance Framework for Health and Social Care and Research Ethics Committee Approval.

Swansea University was the data controller and processor, and we provided training, guidance and practical support to the Bangladesh team, following General Data Protection Regulation (GDPR) guidelines. The project team in Swansea University who collated the data were all trained in GDPR. The only identifiable participant-level information was the signed consent form for the survey which remained in Bangladesh. The paper questionnaires completed in Bangladesh were stored in a locked cabinet owned by one of the Dhaka site

For peer review only

Principal Investigators. The consent forms were held separately to the survey data in a different locked cabinet.

r peer review only

For peer review only - http://bmjopen.bmj.com/site/about/guidelines.xhtml

References

1. Bangladesh Population Available from: https://worldpopulationreview.com/countries/bangladesh-population.

2. Ahmed SM, Alam BB, Anwar I, Begum T, Huque R, Khan JAM, al. e. Bangladesh Health System Review. Pacific ROftW; 2015.

3. Islam MT, Talukder AK, Siddiqui MN, Islam T. Tackling the COVID-19 pandemic: The Bangladesh perspective. J Public Health Res. 2020;9(4):1794. Epub 20201014. doi: 10.4081/jphr.2020.1794. PubMed PMID: 33117758; PubMed Central PMCID: PMCPMC7582102.

4. Asian Development Bank. Poverty Data: Bangladesh. Available from: https://www.adb.org/where-we-work/bangladesh/poverty (Access date 27 November 2023) 2023.

5. Sohrabi C, Alsafi Z, O'Neill N, Khan M, Kerwan A, Al-Jabir A, et al. World Health Organization declares global emergency: A review of the 2019 novel coronavirus (COVID-19). Int J Surg. 2020;76:71-6. Epub 20200226. doi: 10.1016/j.ijsu.2020.02.034. PubMed PMID: 32112977; PubMed Central PMCID: PMCPMC7105032.

6. Bong CL, Brasher C, Chikumba E, McDougall R, Mellin-Olsen J, Enright A. The COVID-19 Pandemic: Effects on Low- and Middle-Income Countries. Anesth Analg. 2020;131(1):86-92. doi: 10.1213/ANE.0000000000004846. PubMed PMID: 32243287; PubMed Central PMCID: PMCPMC7173081.

7. Pasquale S, Gregorio GL, Caterina A, Francesco C, Beatrice PM, Vincenzo P, Caterina PM. COVID-19 in Low- and Middle-Income Countries (LMICs): A Narrative Review from Prevention to Vaccination Strategy. Vaccines (Basel). 2021;9(12). Epub 20211214. doi:

For peer review only

10.3390/vaccines9121477. PubMed PMID: 34960223; PubMed Central PMCID: PMCPMC8704834.

8. H.J. Freudenberger HL. Staff burn-out. 30. 1974;1:159-65.

9. World Health Organisation. Burn-out an "occupational phenomenon": International Classification of Diseases. Available from: https://www.who.int/news/item/28-05-2019-burn-out-an-occupational-phenomenon-international-classification-of-diseases (Access date 27 November 2023) 2019.

10. Saladino V, Auriemma V, Campinoti V. Healthcare Professionals, Post-traumatic Stress Disorder, and COVID-19: A Review of the Literature. Front Psychiatry. 2021;12:795221. Epub 20220121. doi: 10.3389/fpsyt.2021.795221. PubMed PMID: 35126205; PubMed Central PMCID: PMCPMC8813735.

11. Buran F, Altin Z. Burnout among physicians working in a pandemic hospital during the COVID-19 pandemic. Leg Med (Tokyo). 2021;51:101881. Epub 2021/04/16. doi: 10.1016/j.legalmed.2021.101881. PubMed PMID: 33857752; PubMed Central PMCID: PMCPMC8025601.

12. AlJhani S, AlHarbi H, AlJameli S, Hameed L, AlAql K, Alsulaimi M. Burnout and coping among healthcare providers working in Saudi Arabia during the COVID-19 pandemic. Middle East Current Psychiatry. 2021;28(29). doi: https://doi.org/10.1186/s43045-021-00108-6.

13. Sasangohar F, Jones SL, Masud FN, Vahidy FS, Kash BA. Provider Burnout and Fatigue During the COVID-19 Pandemic: Lessons Learned From a High-Volume Intensive Care Unit. Anesth Analg. 2020;131(1):106-11. Epub 2020/04/14. doi: 10.1213/ANE.0000000000004866. PubMed PMID: 32282389; PubMed Central PMCID: PMCPMC7173087.

14. Dillon EC, Stults CD, Deng S, Martinez M, Szwerinski N, Koenig PT, et al. Women, Younger Clinicians', and Caregivers' Experiences of Burnout and Well-being During COVID-19

For peer review only - http://bmjopen.bmj.com/site/about/guidelines.xhtml

in a US Healthcare System. J Gen Intern Med. 2022;37(1):145-53. Epub 2021/11/04. doi: 10.1007/s11606-021-07134-4. PubMed PMID: 34729697; PubMed Central PMCID: PMCPMC8562379.

15. Khan N, Palepu A, Dodek P, Salmon A, Leitch H, Ruzycki S, et al. Cross-sectional survey on physician burnout during the COVID-19 pandemic in Vancouver, Canada: the role of gender, ethnicity and sexual orientation. BMJ Open. 2021;11(5):e050380. Epub 2021/05/12. doi: 10.1136/bmjopen-2021-050380. PubMed PMID: 33972345; PubMed Central PMCID: PMCPMC8111871.

16. Cotel A, Golu F, Pantea Stoian A, Dimitriu M, Socea B, Cirstoveanu C, et al. Predictors of Burnout in Healthcare Workers during the COVID-19 Pandemic. Healthcare (Basel). 2021;9(3). Epub 2021/04/04. doi: 10.3390/healthcare9030304. PubMed PMID: 33803286; PubMed Central PMCID: PMCPMC8001536.

17. Duarte I, Teixeira A, Castro L, Marina S, Ribeiro C, Jacome C, et al. Burnout among Portuguese healthcare workers during the COVID-19 pandemic. BMC Public Health. 2020;20(1):1885. Epub 2020/12/09. doi: 10.1186/s12889-020-09980-z. PubMed PMID: 33287794; PubMed Central PMCID: PMCPMC7720923.

18. Greene T, Harju-Seppänen J, Adeniji M, Steel C, Grey N, Brewin CR, et al. Predictors and rates of PTSD, depression and anxiety in UK frontline health and social care workers during COVID-19. European Journal of Psychotraumatology. 2021;12(1):1882781. doi: 10.1080/20008198.2021.1882781.

19. Ahmed T, Musarrat P, Kabir ZN. Lessons learned from pandemic response to COVID-19 in Bangladesh: NGO-based emergency response framework for low- and middle-income countries. BMC Health Serv Res. 2023;23(1):656. Epub 20230620. doi: 10.1186/s12913-023-09643-w. PubMed PMID: 37340495; PubMed Central PMCID: PMCPMC10283326.

For peer review only - http://bmjopen.bmj.com/site/about/guidelines.xhtml

20. Hossain MI, Mehedi N, Ahmad I, Ali I, Azman A. Psychosocial stress and trauma during the COVID-19 pandemic: Evidence from Bangladesh. Asian Soc Work Policy Rev. 2021;15(2):145-59. Epub 20210412. doi: 10.1111/aswp.12227. PubMed PMID: 35655964; PubMed Central PMCID: PMCPMC8250397.

21. Talukder B, vanLoon GW, Hipel KW, Orbinski J. COVID-19's implications on agri-food systems and human health in Bangladesh. Curr Res Environ Sustain. 2021;3:100033. Epub 20210310. doi: 10.1016/j.crsust.2021.100033. PubMed PMID: 34977606; PubMed Central PMCID: PMCPMC7945872.

22. Saifuzzaman M, Rahman MM, Shetu SF, Moon NN. COVID-19 and Bangladesh: Situation report, comparative analysis, and case study. Current Research in Behavioral Sciences 2021;2.

23. Siam MHB, Hasan MM, Tashrif SM, Rahaman Khan MH, Raheem E, Hossain MS. Insights into the first seven-months of COVID-19 pandemic in Bangladesh: lessons learned from a high-risk country. Heliyon. 2021;7(6):e07385. Epub 20210625. doi: 10.1016/j.heliyon.2021.e07385. PubMed PMID: 34189333; PubMed Central PMCID: PMCPMC8226055.

24. Yasir Arafat SM, Kabir R. Burnout in Physicians: Global and Bangladesh Perspective. EC Psychology and Psychiatry. 2017;2.3:112-7.

25. Chowdhury SR, Kabir H, Mazumder S, Akter N, Chowdhury MR, Hossain A. Workplace violence, bullying, burnout, job satisfaction and their correlation with depression among Bangladeshi nurses: A cross-sectional survey during the COVID-19 pandemic. PLoS One. 2022;17(9):e0274965. Epub 20220922. doi: 10.1371/journal.pone.0274965. PubMed PMID: 36137141; PubMed Central PMCID: PMCPMC9499253.

For peer review only

For peer review only - http://bmjopen.bmj.com/site/about/guidelines.xhtml

26. Biswas M, Hasan MT, Samir N, Alin SI, Homaira N, Hassan MZ, et al. The Prevalence and Associated Factors of Depressive Symptoms Among Medical Students in Bangladesh During the COVID-19 Pandemic: A Cross-Sectional Pilot Study. Front Public Health. 2021;9:811345. Epub 20220131. doi: 10.3389/fpubh.2021.811345. PubMed PMID: 35174136; PubMed Central PMCID: PMCPMC8841836.

27. Zigmond AS, Snaith RP. The hospital anxiety and depression scale. Acta Psychiatr Scand. 1983;67(6):361-70. PubMed PMID: 6880820.

28. Bjelland I, Dahl AA, Haug TT, Neckelmann D. The validity of the Hospital Anxiety and Depression Scale. An updated literature review. J Psychosom Res. 2002;52(2):69-77. doi: 10.1016/s0022-3999(01)00296-3. PubMed PMID: 11832252.

29. Maslach C, Jackson SE. The measurement of experienced burnout. Journal of Occupational Behaviour. 1981;2:99-113.

30. Kalliath TJ, O'Driscoll MP, Gillespie DF, Bluedorn AC. A test of the Maslach burnout inventory in three samples of healthcare professionals Work Stress. 14(1):35-50.

31. Lheureux F, Truchot D, Borteyrou X, Rascle N. The Maslach Burnout Inventory – Human Services Survey (MBI-HSS): factor structure, wording effect and psychometric qualities of known problematic items. Le Travail Humain. 2017;80(2):161-86.

32. Lin CY, Alimoradi Z, Griffiths MD, Pakpour AH. Psychometric properties of the Maslach Burnout Inventory for Medical Personnel (MBI-HSS-MP). Heliyon. 2022;8(2):e08868. Epub 20220201. doi: 10.1016/j.heliyon.2022.e08868. PubMed PMID: 35169645; PubMed Central PMCID: PMCPMC8829575.

33. Brady KJS, Ni P, Sheldrick RC, Trockel MT, Shanafelt TD, Rowe SG, et al. Describing the emotional exhaustion, depersonalization, and low personal accomplishment symptoms associated with Maslach Burnout Inventory subscale scores in US physicians: an item

For peer review only - http://bmjopen.bmj.com/site/about/guidelines.xhtml

response theory analysis. J Patient Rep Outcomes. 2020;4(1):42. Epub 20200601. doi: 10.1186/s41687-020-00204-x. PubMed PMID: 32488344; PubMed Central PMCID: PMCPMC7266903.

34. Maslach C, Jackson SE, Leiter MP. Maslach burnout inventory manual. Menlo Park, CA: Mind Garden Inc, 1996-2008. Report No.

35. Stern AF. The hospital anxiety and depression scale. Occup Med (Lond). 2014;64(5):393-4. doi: 10.1093/occmed/kqu024. PubMed PMID: 25005549.

36. Aly HM, Nemr NA, Kishk RM, Elsaid N. Stress, anxiety and depression among healthcare workers facing COVID-19 pandemic in Egypt: a cross-sectional online-based study. BMJ Open. 2021;11(4):e045281. Epub 2021/05/02. doi: 10.1136/bmjopen-2020-045281. PubMed PMID: 33931409; PubMed Central PMCID: PMCPMC8098284.

37. Ali Sahebi A, Nejati-Zarnaqi B, Moayedi S, Yousefi K, Torres M, Golitaleb M. The prevalence of anxiety and depression among healthcare workers during the COVID-19 pandemic: An umbrella review of meta-analyses. Progress in Neuropsychopharmacology and Biological Psychiatry. 2021;107(110247).

38. Fernandez R, Sikhosana N, Green H, Halcomb EJ, Middleton R, Alananzeh I, et al. Anxiety and depression among healthcare workers during the COVID-19 pandemic: a systematic umbrella review of the global evidence. BMJ Open. 2021;11(9):e054528. Epub 2021/09/23. doi: 10.1136/bmjopen-2021-054528. PubMed PMID: 34548373; PubMed Central PMCID: PMCPMC8458002.

39. Yin C, Ji J, Cao X, Jin H, Ma Q, Gao Y. Impact of long working hours on depressive symptoms among COVID-19 frontline medical staff: The mediation of job burnout and the moderation of family and organizational support. Front Psychol. 2023;14:1084329. Epub

For peer review only - http://bmjopen.bmj.com/site/about/guidelines.xhtml

20230215. doi: 10.3389/fpsyg.2023.1084329. PubMed PMID: 36874813; PubMed Central PMCID: PMCPMC9975257.

40. Bari R, Sultana F. Second Wave of COVID-19 in Bangladesh: An Integrated and Coordinated Set of Actions Is Crucial to Tackle Current Upsurge of Cases and Deaths. Front Public Health. 2021;9:699918. Epub 20210830. doi: 10.3389/fpubh.2021.699918. PubMed PMID: 34527649; PubMed Central PMCID: PMCPMC8437241.

41. Hasan MT, Anwar T, Christopher E, Hossain S, Hossain MM, Koly KN, et al. The current state of mental healthcare in Bangladesh: part 1 - an updated country profile. BJPsych Int. 2021;18(4):78-82. doi: 10.1192/bji.2021.41. PubMed PMID: 34747942; PubMed Central PMCID: PMCPMC8554893.

For peer review only

For peer review only - http://bmjopen.bmj.com/site/about/guidelines.xhtml

Figures

Figure 1. Proportion of participants exhibiting normal or abnormal HADS Anxiety and Depression scores by institution.

Figure 2. Proportion of participants in each institution achieving threshold levels for burnout using the MBI scores for EE, DP and PA.

For peer review only

For peer review only - http://bmjopen.bmj.com/site/about/guidelines.xhtml

Tables

For peer review only

For peer review only - http://bmjopen.bmj.com/site/about/guidelines.xhtml

Table 1: Demographic information by Institution

	Institution			
	**SRNGIH n=400**	**DMCH n=1283**	**MMCH n=628**	**MARMC n=394**
**Age (years)**				
Mean (s.d.)	32.2 (7.3)	33.6 (8.4)	37.1 (8.1)	32.3 (7.4)
**Gender (%)**				
Female: Male	72:28	82:18	71:29	83:17
**Departments (n,%)**				
Internal Medicine	12 (3)	227 (17.7)	99 (15.7)	57 (14.5)
Cardiology	1 (0.3)	1 (0.1)	12 (1.9)	26 (6.6)
Gastroenterology	207 (51.8)	6 (0.5)	9 (1.4)	0
Respiratory Medicine	4 (1.0)	7 (0.6)	8 (1.3)	0
Nephrology	1 (0.3)	55 (4.3)	20 (3.2)	16 (4.1)
Neurology	0	25 (1.9)	2 (0.3)	9 (2.3)
Rheumatology	3 (0.8)	3 (0.2)	5(0.8)	1 (0.3)
Surgery	21 (5.3)	280 (21.8)	69 (11.0)	83 (21.1)
Gynae/Obs	1 (0.3)	124 (9.7)	57 (9.1)	38 (9.6)
Psychiatry	1 (0.3)	8 (0.6)	4 (0.6)	0
Other	149 (37.3)	533 (41.5)	340 (54.1)	156 (39.6)
Nmiss (%)	0	14 (1.1)	3 (0.5)	8 (2.0)
**Professions (n,%)**				
Academic	31 (7.8)	2 (0.2)	119 (18.9)	5 (1.3)
Doctor	89 (22.3)	157 (12.2)	193 (30.7)	76 (19.3)
Nurse	277 (69.3)	1092 (85.1)	311 (49.5)	312 (79.2)
Trainee	3 (0.8)	30 (2.3)	4 (0.6)	1 (0.3)
Nmiss (%)	0	2 (0.2)	1 (0.2)	0
**COVID rota (n,%)**				
Yes	357 (89.3)	490 (38.2)	609 (97.0)	201 (51.0)
No	43 (10.8)	792 (61.7)	19 (3.0)	193 (49.0)
**Govt. Employee**				
Yes	395 (98.8)	1146 (89.3)	624 (99.4)	386 (98.0)
No	5 (1.3)	137 (10.7)	3 (0.5)	8 (2.0)
Nmiss(%)	0	0	1 (0.2)	0
**Private Practice**				
Yes	76 (19.0)	29 (2.3)	117 (18.6)	8 (2.0)
No	324 (81.0)	1252 (97.6)	510 (81.2)	86 (98.0)
**Awareness of a Wellbeing Service**				
Yes	190 (47.5)	606 (47.2)	244 (38.9)	174 (44.2)
No	209 (52.3)	672 (52.4)	383 (60.9)	214 (54.3)
**Wellbeing Service (Getting help) [multiple response]**Friends/Family				
Work Colleagues	20 (10.5)	63 (10.4)	48 (19.7)	4 (2.3)
Occupational Health	93 (49.0)	356 (58.8)	110 (45.1)	120 (69.0)
None	10 (5.3)	15 (2.5)	5 (2.1)	14 (8.1)
Other	66 (34.7)	172 (28.4)	84 (34.4)	38 (21.8)
	2 (1.1)	9 (1.5)	0	0

For peer review only - http://bmjopen.bmj.com/site/about/guidelines.xhtml

Table 2: Hospital Anxiety and Depression Score (HADs) and Maslach Burnout Scores by Institution

	Institutions					
**HADs**	**SRNGIH**	**DMCH**	**MMCH**	**MARMC**	**F-test (*p-value*)**	**Tukey *t-test (significant)*[1]**
**Anxiety Subscale Score**<br>Mean (s.d.)	7.6 (4)	7.8 (4)	8.6 (4.1)	9 (4.3)	14.7 (<0.01)	G1 vs G2<br>G1 vs G3*<br>G1 vs G4*<br>G2 vs G3*<br>G2 vs G4*<br>G3 vs G4
**Depression Subscale Score**<br>Mean (s.d.)	6.3 (4.1)	6.6 (3.7)	7.9 (3.9)	6.9 (4.5)	17.3 (<0.01)	G1 vs G2<br>G1 vs G3*<br>G1 vs G4<br>G2 vs G3*<br>G2 vs G4<br>G3 vs G4*
**Maslach Subscale Scores**	**SRNGIH**	**DMCH**	**MMCH**	**MARMC**	**F-test (*p-value*)**	**Tukey *t-test (significant)*[1]**
**Emotional exhaustion (EE)**<br>Mean (s.d.)	17.5 (11.1)	15.4 (10.0)	19.1 (10.8)	15.7 (8.1)	20.6 (*p<0.01*)	G1 vs G2*<br>G1 vs G3<br>G1 vs G4<br>G2 vs G3*<br>G2 vs G4<br>G3 vs G4*
**Depersonalisation / loss of empathy (DP)**<br>Mean (s.d.)	4.3 (5.3)	4.3 (5.1)	5.2 (5.2)	4.7 (5.8)	4.11 (*p<0.01*)	G1 vs G2<br>G1 vs G3*<br>G1 vs G4<br>G2 vs G3*<br>G2 vs G4<br>G3 vs G4
**Personal accomplishment assessment (PA)**<br>Mean (s.d.)	39.0 (8.4)	38.5 (8.8)	36.5 (9.4)	37.8 (7.4)	8.9 (*p<0.01*)	G1 vs G2<br>G1 vs G3*<br>G1 vs G4<br>G2 vs G3*

For peer review only - http://bmjopen.bmj.com/site/about/guidelines.xhtml

	Institutions					
**HADs**	**SRNGIH**	**DMCH**	**MMCH**	**MARMC**	**F-test (*p-value*)**	**Tukey *t-test (significant)*[1]**
						G2 vs G4<br>G3 vs G4

For peer review only

For peer review only - http://bmjopen.bmj.com/site/about/guidelines.xhtml

Table 3: Hospital Anxiety and Depression Score (HADs) and Maslach Burnout Scores by COVID duty

	COVID Duty		*t*-test (*p*-value)
**HADs**	**Yes**	**No**	
**Anxiety Subscale Score**<br>Mean (s.d.)	8.3 (4.2)	7.8 (3.9)	3.4 (*p<0.01*)
**Depression Subscale Score**<br>Mean (s.d.)	7.1 (4.2)	6.5 (3.7)	4.2 (p<0.01)
**Maslach Subscale Scores**	**Yes**	**No**	**Chi-Square (*p-value*)**
**Emotional exhaustion (OE)**<br>Mean (s.d.)	17.5 (10.4)	15.3 (9.8)	5.4 (p<0.01)
**Depersonalisation / loss of empathy (DP)**<br>Mean (s.d.)	4.9 (5.4)	4.1 (5.0)	3.9 (p<0.01)
**Personal accomplishment assessment (PA)**<br>Mean (s.d.)	37.3 (9.1)	39.2 (8.1)	-5.4 (p<0.01)

For peer review only

For peer review only - http://bmjopen.bmj.com/site/about/guidelines.xhtml

Figure 1. Proportion of participants exhibiting normal or abnormal HADS Anxiety and Depression scores by institution.

a) HADs Anxiety scores

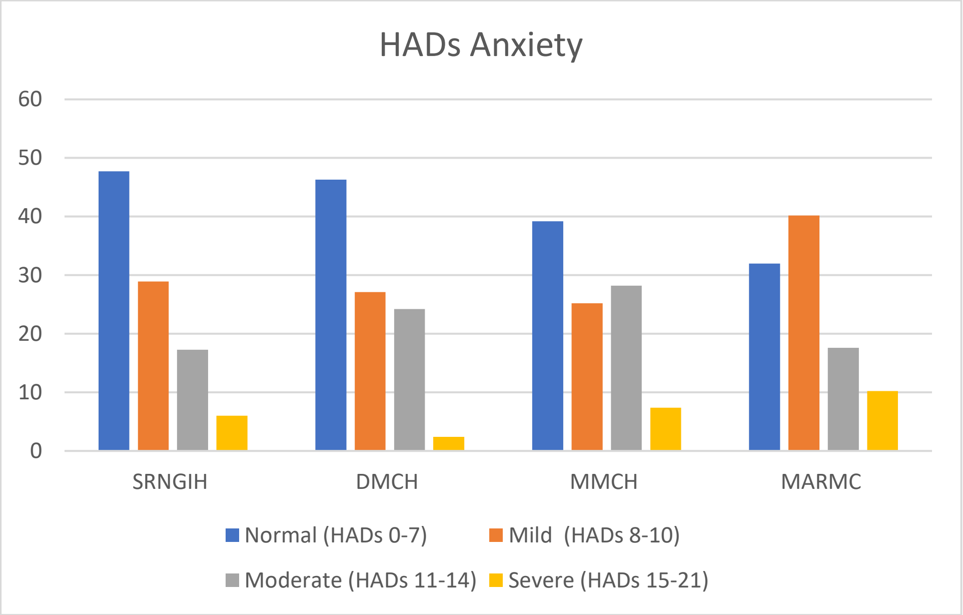

*Statistically significant results (Chi- squared comparison with Bonferroni corrections):
SRNGIH vs DMCH, $p<0.001$; SRNGIH vs MMCH, $p<0.001$; SRNGIH vs MARMC, $p<0.001$
DMCH vs MMCH, $p<0.001$; DMCH vs MARMC, $p<0.001$
MMCH vs MARMC, $p<0.001$

b) HADs Depression scores

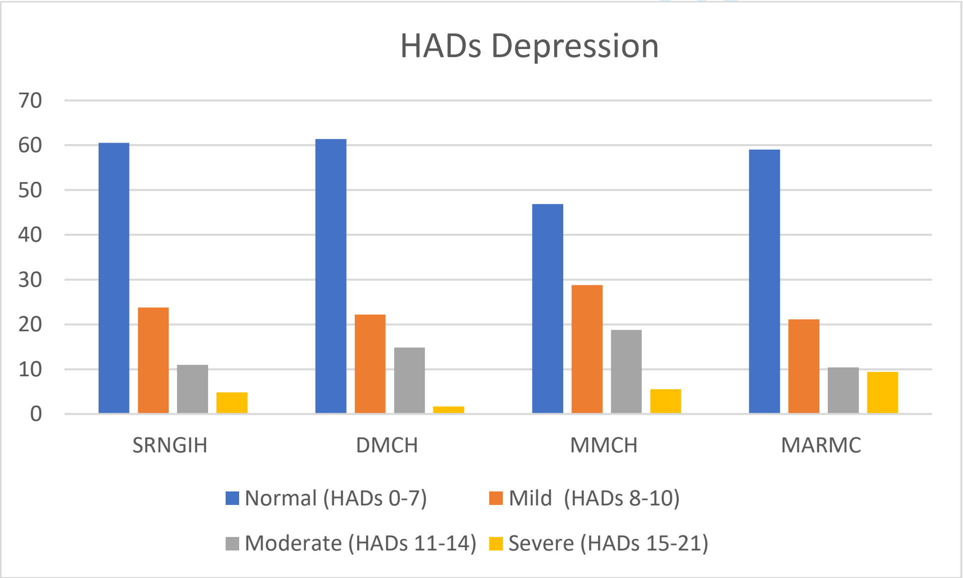

*Statistically significant results (Chi- squared comparison with Bonferroni corrections):
SRNGIH vs DMCH, $p=0.002$; SRNGIH vs MMCH, $p<0.001$
DMCH vs MMCH, $p<0.001$; DMCH vs MARMC, $p<0.001$
MMCH vs MARMC, $p<0.001$

Figure 2. Proportion of participants in each institution achieving threshold levels for burnout using the MBI scores for EE, DP and PA.

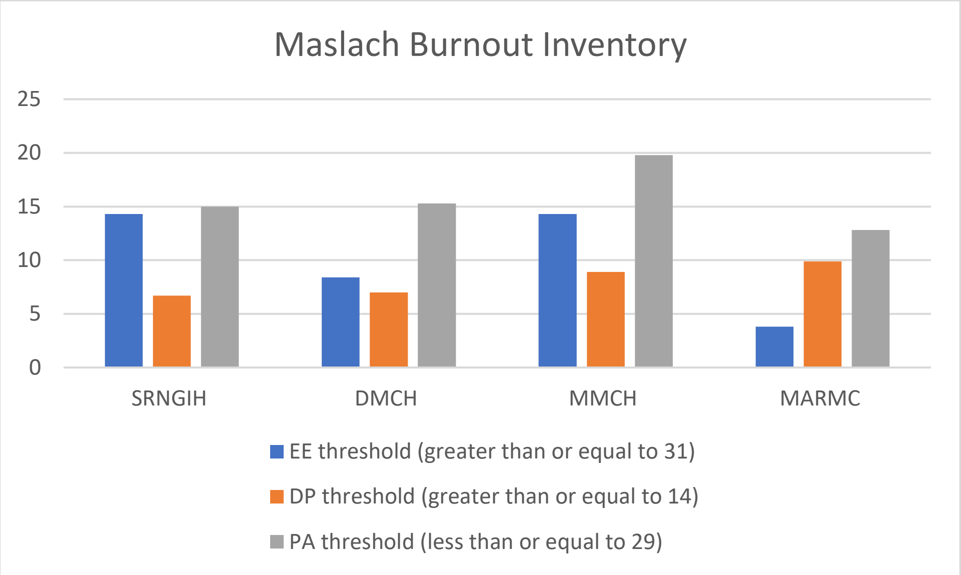

Maslach Burnout Inventory (MBI) %	SRNGIH	DMCH	MMCH	MARMC
EE threshold (greater than or equal to 31)*	14.3	8.4	14.3	3.8
DP threshold (greater than or equal to 14)	6.7	7	8.9	9.9
PA threshold (less than or equal to 29)*	15	15.3	19.8	12.8

*Statistically significant results (Chi- squared comparison with Bonferroni corrections):

EE

SRNGIH vs MARMC, p<0.001

DMCH vs MMCH, p<0.001; DMCH vs MARMC, p<0.002

MMCH vs MARMC, p<0.001

PA

SRNGIH vs MARMC, p<0.005

DMCH vs MMCH, p<0.001; DMCH vs MARMC, p<0.002

MMCH vs MARMC, p<0.004

For peer review only - http://bmjopen.bmj.com/site/about/guidelines.xhtml

Supplementary information Table S1: STROBE checklist for crosse-sectional studies indicating where relevant information is found in the manuscript.

Section	Item no	Recommendation	Page no in manuscript
**Title and abstract**	1	*(a)* Indicate the study's design with a commonly used term in the title or the abstract<br>*(b)* Provide in the abstract an informative and balanced summary of what was done and what was found	1<br>2-3
**Introduction**			
Background/rationale	2	Explain the scientific background and rationale for the investigation being reported	5-6
Objectives	3	State specific objectives, including any prespecified hypotheses	6-7
**Methods**			
Study design	4	Present key elements of study design early in the paper	7
Setting	5	Describe the setting, locations, and relevant dates, including periods of recruitment, exposure, follow-up, and data collection	7-8
Participants	6	(*a*) Give the eligibility criteria, and the sources and methods of selection of participants	7-8
Variables	7	Clearly define all outcomes, exposures, predictors, potential confounders, and effect modifiers. Give diagnostic criteria, if applicable	7-8
Data sources/ measurement	8*	For each variable of interest, give sources of data and details of methods of assessment (measurement). Describe comparability of assessment methods if there is more than one group	7-8
Bias	9	Describe any efforts to address potential sources of bias	8
Study size	10	Explain how the study size was arrived at	7
Quantitative variables	11	Explain how quantitative variables were handled in the analyses. If applicable, describe which groupings were chosen and why	9-10
Statistical methods	12	*(a)* Describe all statistical methods, including those used to control for confounding<br>*(b)* Describe any methods used to examine subgroups and interactions<br>*(c)* Explain how missing data were addressed<br>*(d)* If applicable, describe analytical methods taking account of sampling strategy<br>*(e)* Describe any sensitivity analyses	7-10
**Results**			
Participants	13*	(a) Report numbers of individuals at each stage of study—eg numbers potentially eligible, examined for eligibility, confirmed eligible, included in the study, completing follow-up, and analysed<br>(b) Give reasons for non-participation at each stage<br>(c) Consider use of a flow diagram	10
Descriptive data	14*	(a) Give characteristics of study participants (eg demographic, clinical, social) and information on exposures and potential confounders	10-12

For peer review only

		(b) Indicate number of participants with missing data for each variable of interest	28, Table 1
Outcome data	15*	Report numbers of outcome events or summary measures	10-12<br>28-30<br>Tables 1, 2, 3
Main results	16	*(a)* Give unadjusted estimates and, if applicable, confounder-adjusted estimates and their precision (eg, 95% confidence interval). Make clear which confounders were adjusted for and why they were included<br>*(b)* Report category boundaries when continuous variables were categorized<br>*(c)* If relevant, consider translating estimates of relative risk into absolute risk for a meaningful time period	10-12<br>28-30<br>Tables 1, 2, 3
Other analyses	17	Report other analyses done—eg analyses of subgroups and interactions, and sensitivity analyses	N/A
**Discussion**			
Key results	18	Summarise key results with reference to study objectives	13
Limitations	19	Discuss limitations of the study, taking into account sources of potential bias or imprecision. Discuss both direction and magnitude of any potential bias	14-15
Interpretation	20	Give a cautious overall interpretation of results considering objectives, limitations, multiplicity of analyses, results from similar studies, and other relevant evidence	13-15
Generalisability	21	Discuss the generalisability (external validity) of the study results	13-15
**Other information**			
Funding	22	Give the source of funding and the role of the funders for the present study and, if applicable, for the original study on which the present article is based	16

*Give information separately for exposed and unexposed groups.

For peer review only
